# Synthesis of Chiral Acyclic Pyrimidine Nucleoside Analogues from DHAP-Dependent Aldolases

**DOI:** 10.3390/biom14070750

**Published:** 2024-06-25

**Authors:** Mariano Nigro, Israél Sánchez-Moreno, Raúl Benito-Arenas, Ana L. Valino, Adolfo M. Iribarren, Nicolás Veiga, Eduardo García-Junceda, Elizabeth S. Lewkowicz

**Affiliations:** 1 Laboratorio de Biotransformaciones y Química de Ácidos Nucleicos, Universidad Nacional de Quilmes, Bernal 1876, Argentina; nigromarianoj@gmail.com (M.N.); avalino@unq.edu.ar (A.L.V.); airibarren@unq.edu.ar (A.M.I.); 2 Departamento de Química Bio-Orgánica, Instituto de Química Orgánica General, Consejo Superior de Investigaciones Científicas, 28006 Madrid, Spain; israel.sanchez@csic.es (I.S.-M.); rbenito@iqog.csic.es (R.B.-A.); 3 Química Inorgánica, Departamento Estrella Campos, Facultad de Química, Universidad de la República (UdelaR), Av. Gral. Flores 2124, Montevideo 11800, Uruguay; nveiga@fq.edu.uy

**Keywords:** aldol reaction, biocatalysis, drug design, stereoselectivity

## Abstract

Dihydroxyacetone phosphate (DHAP)-dependent aldolases catalyze the aldol addition of DHAP to a variety of aldehydes and generate compounds with two stereocenters. This reaction is useful to synthesize chiral acyclic nucleosides, which constitute a well-known class of antiviral drugs currently used. In such compounds, the chirality of the aliphatic chain, which mimics the open pentose residue, is crucial for activity. In this work, three DHAP-dependent aldolases: fructose-1,6-biphosphate aldolase from rabbit muscle, rhanmulose-1-phosphate aldolase from *Thermotoga maritima*, and fuculose-1-phosphate aldolase from *Escherichia coli*, were used as biocatalysts. Aldehyde derivatives of thymine and cytosine were used as acceptor substrates, generating new acyclic nucleoside analogues containing two new stereocenters with conversion yields between 70% and 90%. Moreover, structural analyses by molecular docking were carried out to gain insights into the diasteromeric excess observed.

## 1. Introduction

**Figure 1:**
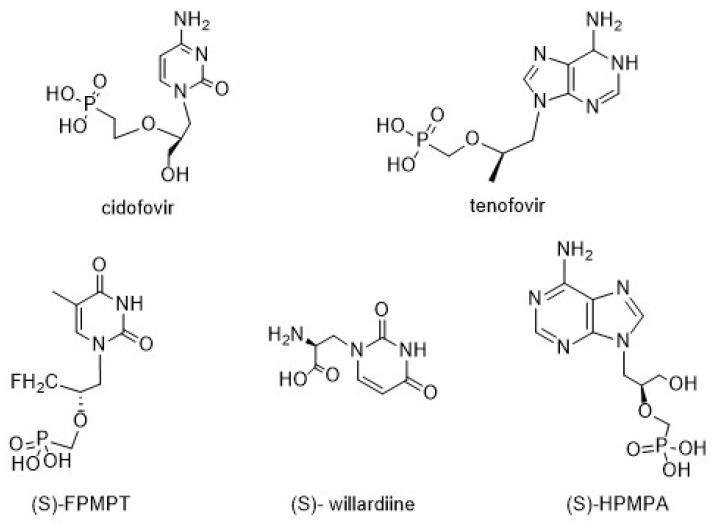
Representative acyclic nucleosides with medicinal activities.

Nucleoside analogues are compounds broadly used as therapeutic agents because they mimic physiological metabolites and interfere in viral and/or cancer cell proliferation [1,2]. In particular, chiral acyclic nucleosides (AN) constitute a well-known nucleoside class that mimics the open pentose residue, and they are currently used as antiviral drugs [3]. (*S*)-Cidofovir, a broad-spectrum antiviral agent, is currently used to treat human cytomegalovirus (HCMV) retinitis related to AIDS [4]. Moreover, 9-[2-(*R*)-(phosphonomethoxy)propyl]adenine (tenofovir), which was approved by the FDA in 2001, has become the most commercially successful drug for the treatment of HIV and HBV infections [5,6,7,8,9]. Some other nucleosides and nucleotides with chiral carbons in the acyclic side chain, such as (*S*)-FPMPT, (*S*)-willardiine, and (*S*)-HPMPA, have different medicinal activities (Figure 1) [10].

Many research efforts have been implemented to carry out synthetic strategies for the preparation of chiral acyclic nucleosides since the configuration of the chiral centers in their aliphatic side chain plays an essential role in their biological activities. Different reactions, such as alkylation, Mitsunobu reaction, epoxide ring-opening, and silylative *N*-hydroxyalkylation, were used for the synthesis of the chiral chain [11,12,13].

However, up to now, the number of bio-catalyzed steps included in AN industrial preparation strategies is limited, and most of them involve the resolution of racemic mixtures obtained by chemical methods [14,15]. During the last years, we have been working on the synthesis of acyclic nucleoside analogues by building the side chain from the corresponding base using bio- or organo-catalyzed aldol addition between 2-oxoethyl derivatives of nucleobases and a suitable ketone [16,17]. The bio-catalyzed strategy involves the use of aldolases, enzymes belonging to the lyases group, which catalyze the stereoselective C-C bonds’ formation by aldol addition between an aldehyde and a ketone [18]. In general, these enzymes show a strict specificity for the electron-donor (commonly a ketone), but they can use a wide range of acceptor aldehydes. Besides, two classes of aldolases can be distinguished depending on the interactions between the donor and the enzyme that originate the true nucleophile of the aldol reaction. Class I aldolases exhibit a lysine residue conserved in the active site that forms an enamine intermediate with the donor, while enzymes belonging to Class II possess a divalent metal ion that promotes enolization of the electron-donor [19,20].

**Figure 2:**
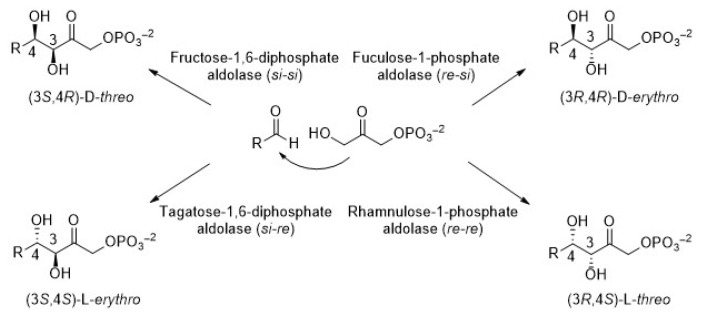
Stereoselective products generated by the four DHAP-dependent aldolases.

In particular, dihydroxyacetone phosphate (DHAP)-dependent aldolases catalyze the aldol addition of DHAP to a variety of aldehydes [21]. As a consequence, compounds containing two new stereocenters are generated. As observed in Figure 2, there are four aldolases that generate products whose stereochemistry at C3 and C4 is complementary. It is widely established that the selectivity of the reaction is completely controlled by the enzyme and not by the substrates. Fructose-1,6-biphosphate aldolase (FDPA) and tagatose-1,6-diphosphate aldolase (TDPA) are mainly Class I aldolases (Class II also exists but only in bacteria, [22]). They use D-glyceraldehyde-3-phosphate (D-G3P) as the natural acceptor substrate to yield D-fructose-1,6-diphosphate and D-tagatose-1,6-diphosphate, respectively. Otherwise, rhanmulose-1-phosphate aldolase (Rhu-1PA) and fuculose-1-phosphate aldolase (Fuc-1PA) naturally catalyze the reversible condensation of DHAP and L-lactaldehyde to afford L-rhanmulose-1-phosphate and L-fuculose-1-phosphate, respectively. Both enzymes belong to the Class II aldolases and require Zn^2+^ as a cofactor, which acts as a Lewis acid in the active site [23,24].

The DHAP-dependent aldolases have been mainly applied to the biosynthesis of carbohydrates and their derivatives [25]. As mentioned above, after the aldol reaction, two new stereocenters are generated and, therefore, by varying the type of DHAP-dependent aldolase and the aldehyde acceptor, a wide range of substituted sugars could be synthesized. A successful synthetic reaction is the aldol addition between DHAP and Cbz-amino-aldehydes (e.g., (*S*)-Cbz-alaninal), catalyzed by FDPA from rabbit muscle (RAMA), and Fuc-1PA and Rhu-1PA, both from *Escherichia coli*, to form amino-polyols, which are precursors of molecules with great therapeutic potential for a broad spectrum of diseases [26,27].

RAMA was the first and most studied and employed Class I DHAP-dependent aldolase [28,29], since RAMA can accept a large number of substrates, including unhindered aliphatic aldehydes, alkyl-heteroatom-substituted aldehydes, monosaccharides, and their derivatives [30]. This broad substrate tolerance, together with its high stereoselectivity ((3*S*,4*R*)-D-*threo* configuration at C3 and C4), allowed its use in the synthesis of a large number of compounds. In the field of carbohydrates, hetero-substituted sugars, deoxy sugars, fluoro sugars, and carbohydrates with up to nine carbon atoms have been prepared [31,32,33].

Rhu-1PA was applied to the synthesis of carbohydrates and other chiral compounds, such as casuarine, an active component from *E. jambolana* and *Eugenia uniflora* used in natural Paraguayan and Indian medicine [27]. Rhu-1PA from *Escherichia coli* was informed for the first time by Fessner et al. in 1991 [34], but this enzyme suffered low operational stability when used as a biocatalyst. To solve this drawback, Rhu-1PAs from thermophilic organisms, such as *Thermotoga maritima*, were preferred for biotechnological applications [35]. Rare sugars, such as D-psicose, D-sorbose, L-tagatose, and L-fructose, were successfully synthesized in multi-enzyme systems (including Rhu-1PA from *T. maritima*) from DL-glycerol-3-phosphate [36]. Recently, it was found that some Rhu-1Pas, such as that from *Bacteroides thetaiotaomicron*, present unusual activity when α-hydroxylated ketones are used. In addition, branched-chain tertiary alcohols could be obtained in excellent yields and stereoselectivity [37].

Fuc-1PA is an essential lyase in L-fucose metabolism in microorganisms. Enzymes from mesophiles and thermophiles [38,39] have been extensively studied and, in the last ten years, enzymes from psychrophilic microorganisms have also been reported [40]. The ability of Fuc-1PA to selectively afford vicinal diols with the anti-configuration has been demonstrated, which is advantageous for the synthesis of rare sugars [41]. Fuc-1PA generates the (3*R*,4*R*)-diol instead of the (3*R*,4*S*) obtained with Rhu-1PA [42]. The absolute configuration at C-3 is conserved in products from both Fuc-1PA and Rhu-1PA; however, with some non-hydroxylated aliphatic aldehydes, the selectivity at C4 can be slightly reduced [34]. New polyhydroxylated benzopyrrolizidines and cyclohexapyrrolizidines were prepared by a chemoenzymatic strategy that included the use of Fuc-1PA variants [43].

Although when carrying out the TDPA-catalyzed aldol addition between DHAP and its natural substrate the configuration obtained is (3*S*,4*S*), up to now, this enzyme has not been synthetically useful because when using another acceptor substrate, the stereoselectivity is lost [24].

As mentioned, DHAP-dependent aldolases exhibit a strict specificity for DHAP. However, this molecule is unstable and very expensive, which makes the preparative application of DHAP-dependent aldol reactions difficult. DHAP can be prepared either chemically or enzymatically [44]. Regarding chemical synthesis, approaches starting from dihydroxyacetone (DHA) dimer provide a stable precursor of DHAP [45,46]. However, these methods are multi-step synthetic routes, which include several purification steps, have high costs, low yields and, in many cases, use toxic reagents. On the other hand, different enzymatic strategies starting from glycerol or DHA have been developed. In the first case, glycerol-3-phosphate was prepared either by phosphorylation of glycerol catalyzed by the phosphatase phytase [47], or by regioselective opening of the *rac*-glycidol epoxide ring with phosphate [48]. DHAP was obtained by the oxidation of the L-glycerol-3-phosphate catalyzed by glycerophosphate oxidase coupled with hydrogen peroxide decomposition by catalase [49]. The phosphorylation of DHA by acid phosphatases, such as that from *Shigella flexneri*, which utilizes pyrophosphate as phosphate donor [50], or by specific kinases and ATP as a phosphate donor, was reported [51,52]. To avoid the use of ATP in stoichiometric amounts and the accumulation of ADP, which is a strong inhibitor of the kinase activity, Sanchez-Moreno et al. developed a useful strategy for DHAP preparation using a recombinant ATP-dependent dihydroxyacetone kinase (DHAK), with the in situ regeneration of ATP [53,54]. These authors also reported a multi-enzyme approach for one-pot C-C bond formation by aldol addition, coupling the just mentioned DHAP synthesis with recombinant DHAP-dependent aldolases [55]. In this way, different ketose-1-phosphates were prepared employing recombinant DHAK from *Citrobacter freundii* for DHA phosphorylation and Rhua-1PA from *Thermotoga maritima* or Fuc-1PA from *E. coli* as catalysts for the aldol addition, with several aldehyde acceptors [56,57].

**Figure 3:**
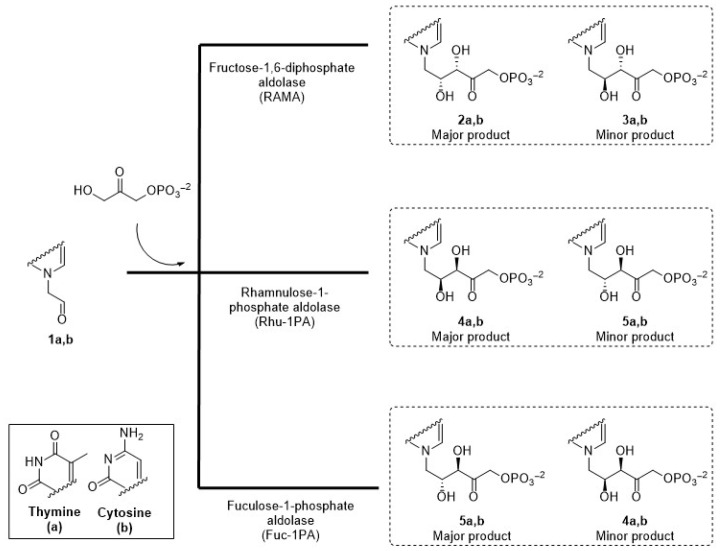
Expected acyclic nucleoside analogues produced by three different aldolases.

In this context, the aim of the present work was to prepare chiral acyclic nucleoside analogues with complementary stereochemistry using three DHAP-dependent aldolases, RAMA, Rhu-1PA from *Thermotoga maritima* (*Tm*Rhu-1PA), and Fuc-1PA from *Escherichia coli* (*Ec*Fuc-1PA), as biocatalysts, and pyrimidyl acetaldehydes as acceptor substrates (Figure 3).

## 2. Materials and Methods

### 2.1. Enzymes and Reagents

*Tm*Rhu-1PA, *Ec*Fuc-1PA, and DHAK from *C. freundii* were cloned, expressed, and purified by the protocols previously described [53]. Thymine, cytosine, 1-bromo-2,2-dimethoxyethane, dihydroxyacetone (DHA), NADH, ATP, fructose-1,6-diphosphate aldolase from rabbit muscle (RAMA), acetate kinase (AK), and α-glycerophosphate dehydrogenase/triose phosphate isomerase (α-GDH/TIM) were purchased from Sigma-Aldrich (St. Louis, MO, USA). PA- and HPLC-grade solvents were from Biopack (Buenos Aires, Argentina), Sintorgan (Buenos Aires, Argentina), or JT Baker (Phillipsburg, NJ, USA). All other chemical reagents were commercially available and of the best analytical grade. Culture media components were obtained from Merck (Darmstadt, Germany) and Difco (Sparks, MD, USA).

### 2.2. Instrumental

HPLC-UV analyses were carried out with a Gilson chromatograph (321 Pump, 156UV/VIS detector, and 234 Autoinjector Series; Middleton, WI, USA). ^1^H and ^13^C NMR spectra were recorded on a Bruker Avance II 500 spectrometer (Madison, WI, USA) at 500 MHz and 125 MHz, respectively, using D_2_O as a solvent. EI-MS spectra were recorded on a Thermo Finnigan LCQ Advantage Max spectrometer (San Jose, CA, USA) by direct injection (4 kV ion-spray voltage and 350 °C capillary temperature), in positive mode. Spectrophotometric assays were performed at 340 nm using a UV-160A-UV-Visible recording Shimadzu spectrophotometer (Kyoto, Japan).

### 2.3. Substrate Synthesis

#### 2.3.1. Aldehydes

Thyminyl and cytosyl acetaldehydes (**1a** and **1b**, respectively) were prepared from the corresponding nucleobases according to our previous report [17]. Thymine or cytosine (3 mmol) and K_2_CO_3_ (6 mmol) were stirred at 50 °C in 30 mL of anhydrous DMF. After 1 h, the temperature was increased to 90 °C, 1-bromo-2,2-dimethoxyethane (6 mmol) was added, and the mixture was stirred for 23 h. The reaction was filtered, the solvent was removed under reduced pressure, and the residue was purified by silica gel column chromatography, affording the corresponding 1-(2,2-dimethoxyethyl)-pyrimidines. Aldehydes **1a** and **1b** were obtained after acid hydrolysis of the corresponding dimethylacetals. NMR and ESI-MS spectral characteristics were the same as those already described [58].

#### 2.3.2. Dihydroxyacetone Phosphate

Phosphorylation of DHA was carried out as previously described [53]. Acetyl phosphate (6 mmol), MgSO_4_ (0.75 mmol), DHA (3 mmol), DHAK (166 U), and AK (180 U) were added to 60 mL of 50 mM phosphate buffer at pH 7.5. The reaction started by addition of ATP (0.1 mmol). Quantification of DHAP was carried out via an enzymatic procedure. This spectrophotometric assay was run at room temperature for 10 min in a final volume of 1 mL, containing 50 mM Tris–HCl buffer, pH 8, NADH (0.2 μmol), the reaction sample (2 μL), and a mixture of α-GDH/TPI (2 μL). For DHAP storage, after the DHAP yield exceeded 95%, pH was adjusted to 5.0 with HCl and was subsequently frozen and lyophilized.

### 2.4. Synthesis of Acyclic Nucleoside Analogues

To 2 mL of reaction mixture comprising freshly prepared aldehydes **1a** or **1b** (20 mM) and DHAP (40 mM) in 20 mM phosphate buffer, pH 6.8, RAMA (10 U), *Tm*Rhu-1PA (10 U), or *Ec*Fuc-1PA (10 U) was then added. The mixtures were stirred at 150 rpm and 30 °C for RAMA and *Ec*Fuc-1PA or 45 °C for *Tm*Rhu-1PA until DHAP disappeared, as verified by the above DHAP spectrophotometric assay. The crude biotransformation was frozen at −20 °C and purified by HPLC. Separation was carried out using a Mediterranean Sea C18 (25 cm × 5.0 cm, particle ∅ = 5 μm), using 20 mM phosphate buffer pH 6, isocratic, as mobile phase, with a flow of 3 mL/min. The purified aldol products (**2–5**) were analyzed by NMR and EM. Overall yields of the products were: from RAMA **2a+3a:** 78% and **2b+3b:** 83%; from *Tm*Rhu-1PA **4a+5a:** 90% and **4b+5b:** 86%; from *Ec*Fuc-1PA **4a+5a:** 90% and **4b+5b:** 76.6%.

### 2.5. ^1^H and ^13^C NMR Product Data

All ^1^H and ^13^C NMR spectra are available in the Supplementary Materials (SM) file.

**2a**

^1^H NMR (D_2_O, 500 MHz) δ (ppm) 7.42 (1H, d, *J* = 1.1 Hz, H-11), 4.67 (1H, dd, *J* = 18.5, 5.4 Hz, H-1*i*), 4.57 (1H, dd, *J* = 18.7, 6.6 Hz, H-1*ii*), 4.49 (1H, d, *J* = 1.9 Hz, H-3), 4.37 (1H, ddd, *J* = 8.9, 4.0, 1.9 Hz, H-4), 3.98 (1H, dd, *J* = 14.3, 4.0 Hz, H-5*i*), 3.81 (1H, dd, *J* = 14.2, 9.0 Hz, H-5*ii*), 1.81 (3H, d, *J* = 0.9 Hz, H-12).

**3a**

^1^H NMR (D_2_O, 500 MHz) δ (ppm) 7.39 (1H, d, *J* = 1.3 Hz, H-11), 4.67 (1H, dd, *J* = 18.5, 5.4 Hz, H-1*i*), 4.57 (1H, dd, *J* = 18.7, 6.6 Hz, H-1*ii*), 4.43 (1H, d, *J* = 6.9 Hz, H-3), 4.11 (1H, m, H-4), 3.92 (1H, dd, *J* = 14.3, 4.1 Hz, 5-H*ii*), 3.75 (dd, *J* = 14.2, 9.0 Hz, 5-H*i*), 1.81 (3H, d, *J* = 1.2 Hz, H-12).

**2a+3a**

^13^C NMR (D_2_O, 125 MHz) δ (ppm): 210.43 (CO, C-2), 167.05 (C, C-9), 152.47 (C, C-7), 143.72 (CH, C-11), 110.45 (C, C-10), 75.70 (CH2, C-1), 68.71 (CHOH, C-3), 68.02 (CHOH, C-4), 50.84 (CH2, C-5), 11.28 (CH3, C-12).

**2b**

^1^H NMR (D_2_O, 500 MHz,) δ (ppm) 7.55 (1H, d, *J* = 7.4 Hz, H-11), 5.96 (1H, d, *J* = 7.3 Hz, H-10), 4.65 (2H, dd, *J* = 18.6, 7.2 Hz, H-1*i*, *H-1ii*), 4.48 (1H, d, *J* = 2.0 Hz, H-3), 4.37 (1H, ddd, *J* = 8.2, 3.6, 1.5 Hz, H-4), 4.06 (1H, dd, *J* = 14.0, 4.0 Hz, H-5*i*), 3.78 (1H, dd, *J* = 14.3, 9.3 Hz, H-5*ii*).

**3b**

^1^H NMR (D_2_O, 500 MHz) δ (ppm) 7.44 (1H, d, *J* = 7.3 Hz, H-11), 5.88 (1H, d, *J* = 7.3 Hz, H-10), 4.65 (2H, dd, *J* = 18.6, 7.2 Hz, H-1*i*, H-1*ii*), 4.44 (1H, d, *J* = 3.2 Hz, H-3), 4.14 (1H, m, H-4), 3.93 (1H, dd, *J* = 14.0, 4.0 Hz, H-5*ii*), 3.59 (1H, dd, *J* = 15.1, 9.3 Hz, H-5*i*).

**2b+3b**

^13^C NMR (D_2_O, 125 MHz,) δ (ppm) 209.75 (CO, C-2), 165.69 (C, C-7), 148.40 C, C-9), 148.03 (CH, C-11), 95.38 (CH, C-10), 75.80 (CHOH, C-3), 68.80 (CHOH, C-4), 68.34 (CH2, C-1), 52.38 (CH2, C-5).

**4a**

^1^H NMR (D_2_O, 500 MHz) δ (ppm) 7.31 (1H, d, *J* = 0.9 Hz, H-11), 4.57 (2H, dd, *J* = 18.7, 7.4 Hz, H-1*i*, H-1*ii*), 4.38 (1H, d, *J* = 1.7 Hz, H-3), 4.19 (1H, ddd, *J* = 8.8, 4.2, 1.8 Hz, H-4), 3.82 (1H, dd, *J* = 14.3, 4.2 Hz, H-5*ii*), 3.72 (1H, dd, *J* = 14.3, 8.9 Hz, H-5*i*), 1.68 (3H, d, *J* = 0.8 Hz, H-12).

**5a**

^1^H NMR (D_2_O, 500 MHz) δ (ppm) 7.26 (1H, d, *J* = 0.9 Hz, H-11), 4.57 (2H, dd, *J* = 18.7, 7.4 Hz, H-1*i*, H-1*ii*), 4.33 (1H, d, *J* = 5.4 Hz, H-3), 4.01 (1H, ddd, *J* = 9.1, 5.2, 4.3 Hz, H-4), 3.60 (1H, dd, *J* = 14.5, 9.1 Hz, H-5*ii*), 3.48 (1H, dd, *J* = 14.0, 3.7 Hz, H-5*i*), 1.68 (3H, d, *J* = 0.8 Hz, H-12).

**4a+5a**

^13^C NMR (D_2_O, 125 MHz) δ (ppm) 210.02 (CO, C-2), 174.90 (C, C-7), 166.94 (C, C-9), 152.42 (CH, C-11), 110.62 (C, C-10), 75.59 (CHOH, C-3), 75.01 (CHOH, C-4), 68.81 (CH2, C-1), 51.65 (CH2, C-5), 11.56 (CH3, C-12).

**4b**

^1^H NMR (D_2_O, 500 MHz) δ (ppm) 7.52 (1H, d, *J* = 7.4 Hz, H-11), 5.95 (1H, d, *J* = 7.4 Hz, H-10), 4.64 (2H, dd, *J* = 18.7, 7.3 Hz, H-1*i*, H-1*ii*), 4.60 (1H, dd, *J* = 18.7, 7.3 Hz, H-1*ii*), 4.41 (1H, d, *J* = 1.9 Hz, H-3), 4.28 (1H, ddd, *J* = 9.0, 3.9, 2.0 Hz, H-4), 3.99 (1H, dd, *J* = 14.1, 4.0 Hz, H-5*ii*), 3.73 (1H, dd, *J* = 14.1, 9.0 Hz, H-5*i*).

**5b**

^1^H NMR (D_2_O, 500 MHz) δ (ppm) 7.50 (1H, d, *J* = 7.4 Hz, H-11), 5.94 (1H, d, *J* = 6.9 Hz, H-10), 4.64 (1H, dd, *J* = 18.7, 7.3 Hz, H-1*i*), 4.56 (1H, dd, *J* = 18.7, 7.3 Hz, H-1*ii*), 4.37 (1H, d, *J* = 4.7 Hz, H-3), 4.09 (1H, ddd, *J* = 8.8, 5.4, 3.2 Hz, H-4), 3.96 (1H, dd, *J*= 14.2, 3.2, H-5*i*), 3.62 (1H, dd, *J* = 14.3, 8.9 Hz, H5*ii*).

**4b+5b**

^13^C-NMR (D_2_O, 125 MHz) δ (ppm): 209.88 (CO, C-2), 164.05 (CO, C-7), 155.41 (C, C-9), 148.05 (CH, C-11), 95.17 (CH, C-10), 75.37 (CHOH, C-3), 68.81 (CHOH, C-4), 68.43 (CH2, C-1), 51.12 (CH2, C-5).

### 2.6. ESI-MS Experiments

**2a+3a, 4a+5a** *m*/*z* found: 338.76, estimated [C_10_H_15_N_2_O_9_P]^+^: 338.08

**2b+3b, 4b+5b** *m*/*z* found: 324.11, estimated [C_9_H_14_N_3_O_8_P]^+^: 323.05

### 2.7. Molecular Docking

The crystal structures of the biological assemblies of *Tm*Rhu-1PA (UniProtKB Q9X0G1) and *Ec*Fuc-1PA (UniProtKB P0AB87) were extracted from the Protein Data Bank (PDB; identifiers 1PVT and 4FUA, respectively). In both cases, the assignment of ionization states and hydrogen coordinates was performed with the *Protonate 3D* module included in the software MOE 2020.22 [59]. The crystallized inhibitor and all the water molecules not bound to it were removed. For the *Tm*Rhu-1PA receptor, additional preparation steps were needed, since no metal center was found in the original PDB file. The cobalt ion coordination site was located by analogy with the structure of the zinc(II)-dependent Rhu-1PA from *E. coli* (1GT7), which was computationally studied by Veiga’s group [60]. From this information, the coordinating histidine residues (His125, His127, and His194) were redirected manually to link them to the Co^2+^ ion. The molecular geometries of the substrates were optimized in gas phase at the PM3 level with the program Hyperchem [61].

The molecular docking calculations were carried out using the GOLD docking program (v 4.1.2) [62], selecting one active site shared by two neighboring protein subunits. As a first step, a flexible model of the DHAP molecule was docked into the active sites of both receptors, keeping the axial water molecule coordinated to the metal center to fulfil the sixth coordinating position. In order to adequately model the coordination of DHAP to the metal center, the distance between the donor atoms and the Zn^2+^/Co^2+^ ion was constrained to lie between 2.0 and 2.2 Å (*k* = 10,000), according to the analogous O-Zn^2+^ distances crystallographically determined for PGH (2.13–2.16 Å) [63]. In all of the docking runs, the binding site was defined as a 10 Å sphere around the metal ion. In addition, to provide flexibility to the protein, some side chains in the binding site were left free to rotate, while others were constrained to specific rotamers: *Tm*Rhu-1PA flexible residues = Asn29, Thr100, Phe167, Ser101, Glu102, Ile156′, Phe157′; *Tm*Rhu-1PA residues with rotamers (number) = Asn26 (9), Leu132 (5), and Glu153′ (4); *Ec*FucA-1PA flexible residues = Ser72, Glu73, Phe131, Tyr113′, Val202′, and Phe206′; *Ec*Fuc-1PA residues with rotamers (number) = Asn29 (3), Ser71 (5), and Met114′ (7). The criteria for this selection were based on the spatial availability around the residues. Free rotation was permitted in cases where the side chains had sufficient space to rotate without significant steric hindrance. Conversely, for residues in more confined environments, specific rotamers that minimized steric clashes were selected. DHAP was docked 40 times, and the docking modes that achieved the highest Goldscore fitness were retained and compared. Other miscellaneous parameters were assigned the default values provided by the GOLD program.

Following the same procedure, flexible models of **1a** and **1b** were docked 100 times using the DHAP-loaded aldolases as receptors. The results were rendered with Discovery Studio Visualizer [64].

## 3. Results

### 3.1. 5-(1-Thyminyl)-3,4-dihydroxy-2-oxopentyl Phosphate and 5-(1-Cytosyl)-3,4-dihydroxy-2-oxopentyl Phosphate Synthesis using RAMA as a Biocatalyst

For more than 20 years, our laboratory has been dedicated to the synthesis of nucleoside analogues using bio-catalyzed strategies and, in particular, during the last 10 years, we have directed our research toward the field of acyclic nucleosides (AN). The chemoenzymatic approach we developed begins with the chemical preparation of the 2-oxoethyl derivatives of nucleobases. These compounds are subsequently used as acceptor substrates, together with the appropriate donor, in the aldol addition bio-catalyzed by commercially available aldolases. Since aldolases are extremely specific for their corresponding donors, sets of acyclic nucleosides with specific alkyl chains can be constructed. Thereby, we prepared novel acyclic nucleosides carrying a 4-hydroxy-2-oxo butyrate skeleton by means of *N*-acetylneuraminic acid aldolase (NeuAcA) from *Clostridium perfringens*, a pyruvate-dependent aldolase [65]. We also used RAMA, the fructose-1,6-diphosphate aldolase from rabbit muscle, which is the most well-known DHAP-dependent aldolase, to obtain 5-(1-thyminyl)-3,4-dihydroxy-2-oxopentyl phosphate [17]. In contrast to NeuAcA, whose stereoselectivity for the aldol addition is strict only with natural substrates, DHAP-dependent aldolases have a high level of stereo-control at the formed chiral centers, with their configurations being highly predictable. In fact, the AN analogues prepared using RAMA as a biocatalyst showed the usual (3*S*,4*R*) stereochemistry (Figure 2) as a consequence of the nucleophilic attack of the enamine intermediate formed between DHAP and the enzyme on the *si* face of the aldehyde acceptor [19]. Additionally, the AN obtained from DHAP-dependent aldolases contain a phosphate group in the acyclic chain, which favors their use as prodrugs [66].

**Figure 4:**
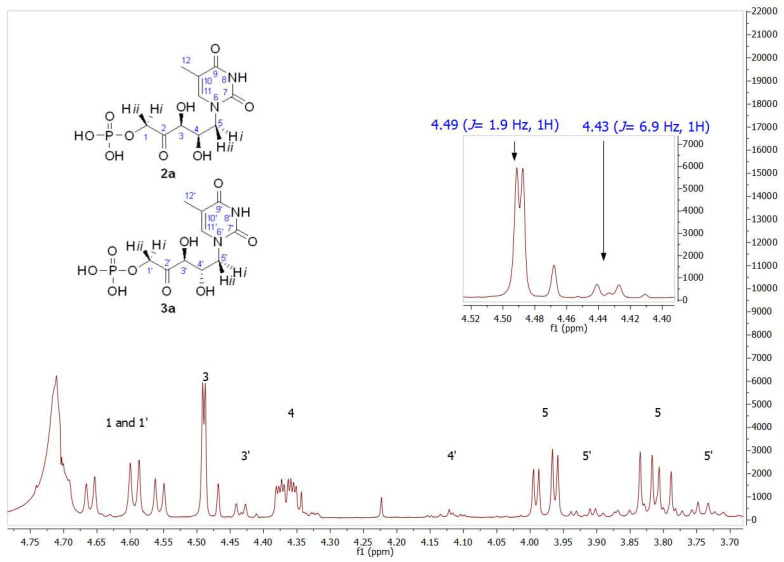
Partial view of the ^1^H NMR spectrum of the aldol product (**2a+3a**) obtained by RAMA bio-catalyzed aldol addition with thyminyl acetaldehyde and DHAP as substrates. The inset shows the different coupling constants of C3 hydrogens (*J*_3–4_ = 1.9 Hz) and C3′ hydrogens (*J*_3′–4′_ = 6.9 Hz), indicating **2a** and **3a** presence, respectively.

As mentioned, the first studies were carried out with the thymine derivative. The aldehyde **1a** (Figure 3) was incorporated into a phosphate-buffered solution containing commercially available DHAP and RAMA and stirred at room temperature. It was necessary to maintain the pH close to 7.0 to ensure the highest efficiency of the enzyme and to avoid the chemical decomposition that DHAP undergoes at higher pH [54]. Conversion to **2a** was followed and quantified by HPLC, and the DHAP consumption was measured by the α-GDH/TIM assay [53]. After HPLC purification, **2a+3a** was obtained in 78% yield. The structure was analyzed by NMR and the molecular weight was confirmed by ESI-MS. ^1^H and ^13^C NMRs of the purified aldols **2a+3a** showed high diastereomeric purity (*d.e.* 97%; Figures S1 and S2). The major signals correspond to the expected *syn*-configuration of the hydroxyl groups in the new chiral centers (**2a**), as explained below. In the ^1^H NMR partial view of **2a+3a** (Figure 4), two double doublets corresponding to the diastereotopic methylenes 1 and 5 were observed. The C5 hydrogens were coupled with the C4 hydrogen, while the C1 hydrogens were coupled with the phosphorus of the phosphate group. In the case of the two C5 hydrogens, both coupling constants *J*_4–5_ were different (4.0 Hz for the 3.98 ppm signal and 9.0 Hz for the 3.81 ppm signal), which would indicate *syn*- and *anti*-configuration between the C4 hydrogen and each C5 hydrogen, H-5*i* and H-5*ii*, respectively, confirming the chirality of C4. On the other hand, the coupling constants between each C1 diastereotopic hydrogen, H-1*i* and H-1*ii*, and the phosphorus of the phosphate group were very similar. The C3 hydrogen appeared as a doublet by coupling with the C4 hydrogen, with *J*_3–4_ = 1.9 Hz, and the double doublet at 4.37 ppm of the C4 hydrogen was the result of the coupling with C5 and C3 hydrogens (9.4 and 1.9 Hz, respectively).

**Figure 5:**
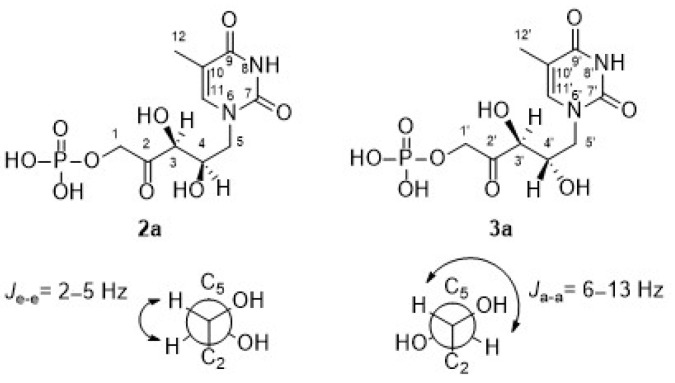
Coupling constants between neighboring protons [67].

Regarding the signal corresponding to C3′ hydrogen of the minor component **3a**, it is noteworthy that it appeared as a doublet at 4.43 ppm, showing a *J*_3′–4′_ = 6.9 Hz (inset in Figure 4). In addition, the *J*_3–4_ = 1.9 Hz of the signal at 4.49 ppm in **2a** is consistent with the coupling dihedral angle value close to 90° (Figure 5), which allows to confirm the D-*threo* configuration expected for the hydroxyl groups of the preferred product of the biotransformation with RAMA, and the L-*erythro* configuration in **3a**.

^13^C NMR (Figure S2) showed the typical signals of the aromatic ring as well as the methyl group, and it can be seen that the signals of carbons 1 to 5 agreed with what was expected.

These results were validated by the group of Dr. Sarotti from Universidad Nacional de Rosario, Argentina, who developed a methodology based on DP4+ probability calculations [68,69]. This updated version of Goodman’s DP4 [70] is used to determine the most likely structure among two or more candidates when one set of experimental data is available. This methodology synergistically combines NMR calculations at higher levels of theory with the Bayesian analysis of both scaled and unscaled data to solve controversial natural products. The DP4+ equation was defined as a function of the corresponding probabilities computed from both scaled (*s*DP4+ term, analogous to the DP4 definition) and unscaled (*u*DP4+ term) chemical shifts. As the unscaled errors did not follow *t* distributions (key basis in subsequent Bayesian analysis), the data were deconvoluted in two *t*-distributed series depending on the hybridization type. In this work, the DP4+ methodology was used for the stereo assignment of the two possible diastereoisomers shown in Figure 5. An exhaustive conformational search was carried out on each isomer at the B3LYP/6-31G* level (gas phase), finding more than 100 conformations for each candidate. Magnetic tensors (σ) were calculated using the GIAO (gauge-including atomic orbitals) [71,72] method at the PCM/mPW1PW91/6-31+G** level [73]. With the calculated tensors and the experimental data, the DP4+ probability was calculated. Therefore, in all cases, the D-*threo* isomer (**2a**) had the highest probability associated, suggesting that it was the correct structure (Table S1).

A similar reaction profile was observed when the cytosine derivative, 5-(1-cytosyl)-3,4-dihydroxy-2-oxopentyl phosphate (**2b**), was prepared. HPLC analysis showed 83% of conversion, and ^1^H NMR (Figure S3) confirmed the expected D-*threo* configuration with 96% *d.e.* Moreover, ^13^C NMR (Figure S4) showed the typical signals of the aromatic ring, and it can be seen that the signals of carbons 1 to 5 were in agreement with those expected.

### 3.2. 5-(1-Thyminyl)-3,4-dihydroxy-2-oxopentyl Phosphate and 5-(1-Cytosyl)-3,4-dihydroxy-2-oxopentyl Phosphatesynthesis Using TmRhu-1PA as a Biocatalyst

With the aim of searching for stereochemical diversity of AN, recombinant Rhu-1PA from *Thermotoga maritima* (*Tm*Rhu-1PA) was first tested [35,54].

Rhu-1PA belongs to Class II aldolases and catalyzes the reversible asymmetric aldol addition between DHAP and L-lactaldehyde, leading to L-rhamnulose-1-phosphate. In the synthetic direction, Rhu-1PA is very specific for DHAP as a donor substrate but accepts a wide range of aldehydes, which has been exploited for synthetic applications [56]. A simple and inexpensive synthesis of unnatural heterocycles, using a one-pot, four-enzyme catalytic cascade, including Rhu-1PA in both free and immobilized forms, has been previously reported [74,75]. Regarding the stereochemistry, a major product was obtained with (3*R*,4*S*, L-*threo*) configuration.

Taking advantage of the hyper-thermophilic origin of *T. maritima* and, therefore, the resistance to high temperatures of its proteins, *Tm*Rhu-1PA purification, carried out at 80 °C, was fast, easy to scale up, and had a high degree of purity. In addition, the purification was performed in the presence of cobalt salts since *Tm*Rhu-1PA remains in solution while most of the other proteins precipitate.

Similar to the study performed with RAMA, *Tm*Rhu-1PA was tested as a biocatalyst for the aldol addition reaction using DHAP and aldehydes **1a** and **1b** as substrates. As mentioned above, it was expected to obtain products **4a** and **4b** with (3*R*,4*S*, L-*threo*) stereochemistry, the enantiomer of the FBPA product (3*S*,4*R*, D-*threo*), previously obtained by RAMA.

**Figure 6:**
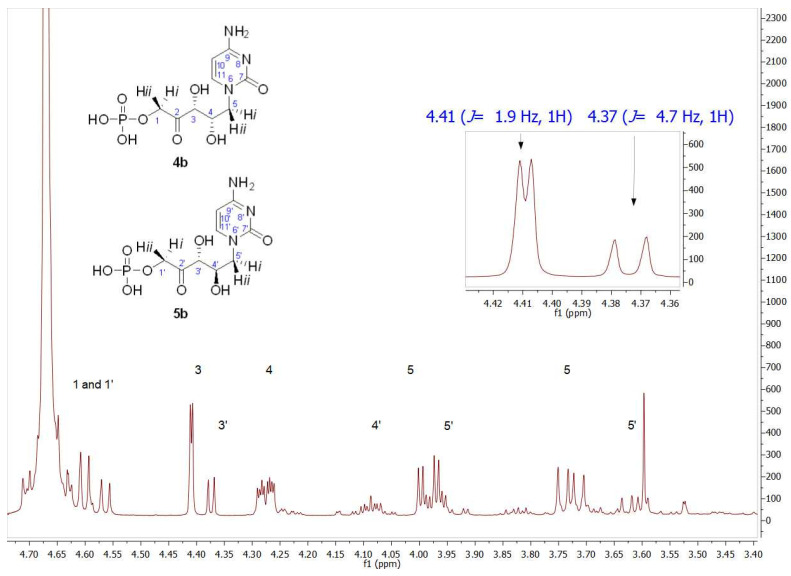
Partial view of the ^1^H NMR spectrum of the aldol product (**4b**+**5b**) obtained by *Tm*Rhua-1PA bio-catalyzed aldol addition, with **1b** and DHAP as substrates. The inset shows C3 hydrogen (*J*_3–4_ = 1.9 Hz) and C3′ hydrogen (*J*_3′–4′_ = 4.7 Hz) signals, indicating **4b** and **5b** presence, respectively.

The formation of the expected **4b** was followed and quantified by HPLC, obtaining 86% conversion. After purification, its structure was analyzed by NMR (Figure S5 for ^1^H NMR and Figure S6 for ^13^C NMR). The structural determination performed by ^1^H NMR showed the presence of two groups of signals with the same profile in an approximate ratio of 75:25. The inset in the ^1^H NMR of Figure 6 shows, paying special attention to the C3 hydrogen, that the signals of the major product were in agreement with those of the aforementioned **2b**, the product previously obtained with RAMA. This result was not surprising since **4b** should be the enantiomer of **2b** and, as it is known, enantiomers are indistinguishable by NMR. Regarding the signal corresponding to C3′ hydrogen of the minor component (δ 4.37 ppm, *J*_3′–4′_ = 4.7 Hz), both coupling constants and chemical shift values indicated that this product corresponds to the D-*erythro* isomer, as was described for the observed minor product in RAMA bio-catalyzed biotransformation. Since, as mentioned previously, the C3 configuration is fixed by the interaction of DHAP with the active site of the enzyme, the D-*erythro* isomer would have (3*R*,4*R*) configuration, which corresponds to compound **5b**.

**Figure 7:**
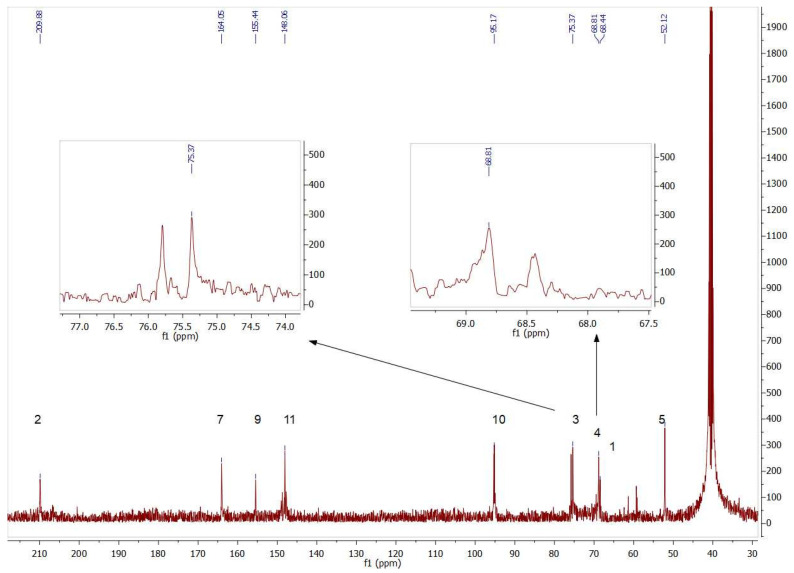
Partial view of ^13^C NMR spectrum of the aldol products (**4b**+**5b**) obtained by*Tm*Rhua-1PA bio-catalyzed aldol addition and **1b** as a substrate. The insets show the double signals corresponding to the chiral carbons C3 and C4, confirming the presence of both **4b** and **5b** isomers.

The presence of both isomers could also be seen by analyzing the ^13^C NMR signals. Figure 7 contains insets of the signals corresponding to the chiral carbons (C3 and C4) of compounds **4b** and **5b**.

Due to the fact that RhuA belongs to Class II aldolases, a c*is*-enediolate was formed by the abstraction of the DHAP C3 pro-R proton, achieving a strict 3*R* stereochemistry. Next, the *re* face of this intermediate was exposed to the electrophile on the *re* face of RhuA, resulting in a 4S configuration [23]. However, depending on the structure of the aldehydes, reverse binding could be observed, leading to a loss of stereoselectivity at C4 [34]. For these reasons, the stereoselectivity observed for *Tm*Rhu-1PA fell within what was expected and, in fact, it had already been seen in previous studies by Sánchez-Moreno et al. [55]. When they used *Tm*Rhu-1PA and benzyloxyacetaldehyde as acceptor substrates, 86% conversion and an 80:20 (3*R*,4*S*, L-*threo*):(3*R*,4*R*, D-*erythro*) diasteromeric ratio were achieved, with the most abundant being the natural isomer for the enzyme.

Similar conditions were used to carry out the biotransformation with the thymine derivative (**1a**) and *Tm*Rhu-1PA as a biocatalyst. The maximum conversion was reached after 90 min of reaction (90%). Once purified, the reaction products were analyzed by NMR (Figure S7 for ^1^H NMR and Figure S8 for ^13^C NMR), observing the same product profile, but in this case the diasteromeric (3*R*,4*S*, L-*threo*):(3*R*,4*R*, *D-erythro*) ratio (**4a** and **5a**) was 80:20.

### 3.3. 5-(1-Thyminyl)-3,4-dihydroxy-2-oxopentyl Phosphate and 5-(1-Cytosyl)-3,4-dihydroxy-2-oxopentyl Phosphate Synthesis Using EcFuc-1PA as a Biocatalyst

Fuc-1PA, as mentioned above, is a Class II DHAP-dependent aldolase, which catalyzes the aldol addition between DHAP and a wide range of acceptor aldehydes, leading to products with two new stereogenic centers with (3*R*,4*R*, D-*erythro*) configuration. Particularly, Fuc-1PA from *E. coli* is a homotetramer, and each of its four active centers are composed of residues from two adjacent subunits, containing one Zn^2+^ ion per subunit [76]. Mechanistically, in the first step, a c*is*-enediolate is formed by the abstraction of the DHAP C3 pro-R proton, achieving a strict 3*R* stereochemistry. Next, the *re* face of this intermediate is exposed to the electrophile on the *si* face, resulting in 4*R* configuration [23]. However, depending on the structure of the aldehydes, reverse binding could be observed, leading to a loss of stereoselectivity at C4 [34].

*Ec*Fuc-1PA was tested as a biocatalyst in the aldol addition of **1a** and **1b** with DHAP. Since *Ec*Fuc-1PA is not a thermostable enzyme, the biotransformation was performed at 30 °C instead of 45 °C, the temperature used when *Tm*Rhu-1PA was the biocatalyst. When **1a** was used as the acceptor aldehyde, 90% conversion was obtained after a 1.5 h reaction, while starting from **1b**, the conversion was 76.6% after a 1 h reaction. The structural analysis of the reaction products was carried out by NMR (Figures S9–S12). Two products were again observed, with the diastereomeric ratios being 75:25 when **1a** was the substrate and 70:30 when **1b** was used as the acceptor aldehyde.

**Figure 8:**
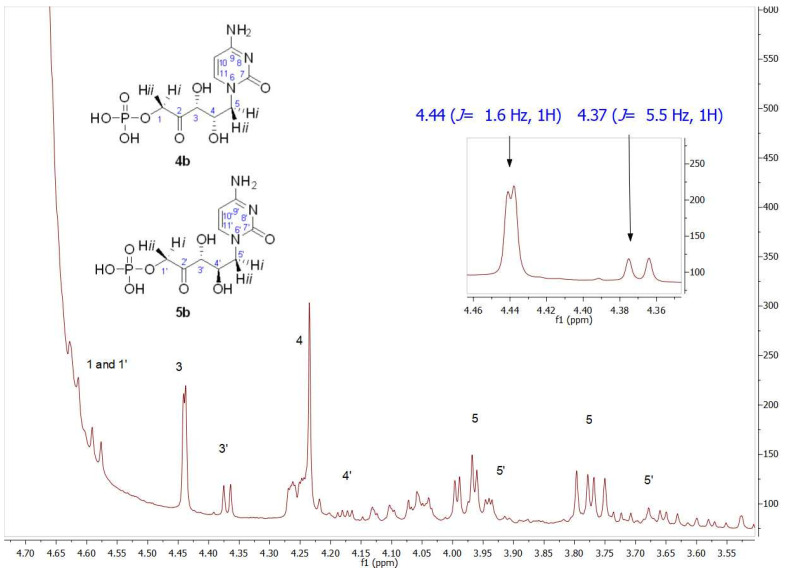
Partial view of ^1^H NMR spectrum of the aldol product (**4b**+**5b**) obtained by *Ec*Fuc-1PA bio-catalyzed aldol addition of **1b** and DHAP as substrates. The inset shows the C3 (*J*_3–4_ = 1.6 Hz) and C3′ (*J*_3′–4′_ = 5.5 Hz) hydrogen signals.

Figure 8 shows a partial view of the ^1^HNMR spectrum after *Ec*Fuc-1PA biotransformation using **1b** as a substrate. Contrary to expectations, the signal profile was similar to that obtained after *Tm*Rhu-1PA biotransformation. Through the analysis of the chemical shifts and the coupling constants, in particular of the C3 and C4 hydrogens, it was evident that, once again, **4b** and **5b** were the major and minor products, respectively. Similar behavior was also observed from **1a.** Although it is not very common for this to happen, the inversion of the expected diastereoselectivity for *Ec*Fuc-1PA, it is not the first time it has been described. Espelt et al. [77] reported the aldol reaction of *N*-Cbz-amino aldehydes and other *N*-protected-3-aminopropanal derivatives with DHAP as substrates and Fuc-1PA as a biocatalyst in emulsion systems. In these works, the stereochemistry of the obtained aldols using both *Tm*Rhu-1PA and *Ec*Fuc-1PA was conserved, affording a (3*R*,4*S*, L-*threo*):(3*R*,4*R*, D-*erythro*) ratio of approximately 80:20 for Rhu-1PA and 67:33 for Fuc-1PA.

### 3.4. Molecular Docking

With the aim of finding an explanation for the behavior of *Ec*Fuc-1A as a biocatalyst in the aldol addition of the pyrimidyl acetaldehyde substrates used in our work, a structural analysis by molecular docking was carried out. Rhu-1PA and Fuc-1PA are known to share several amino acid residues, including the most important active center residues and, in addition, they present a consensus in the central sheet and surrounding helices. Therefore, these enzymes not only have similar chain folds but also similar quaternary structures, and this could be the reason why the C4 stereochemistry is maintained for both enzymes when aldehydes **1a** and **1b** are used as substrates [63].

**Figure 9:**
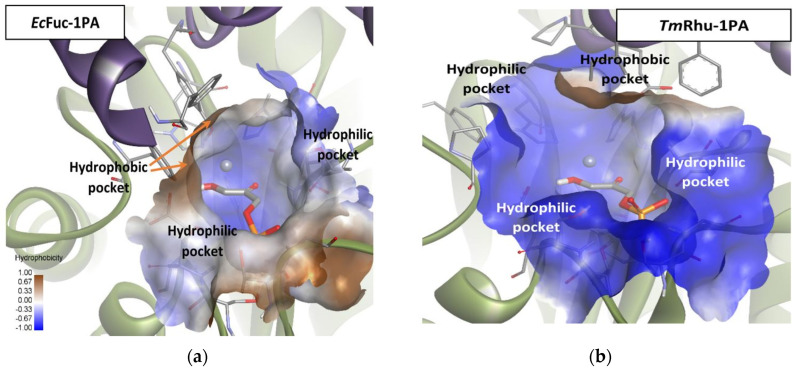
Comparison between the active sites of two DHAP-dependent aldolases: (**a**) DHAP anchored in the *Ec*Fuc-1PA active site and (**b**) DHAP anchored in the *Tm*Rhu-1PA active site. Hydrophobicity is mapped onto a Connolly solvent-accessible surface of the receptor. Non-polar hydrogen atoms are omitted for clarity. Atom color code: C (grey), N (blue), O (red), Zn/Co (violet), P (orange), and H (white). The two protein chains are depicted with different colors (green and turquoise).

Mechanistically, as mentioned before, Fuc-1PA and Rhu-1PA share the stereochemistry of C3, resulting from the interaction of DHAP with the active center of the enzyme. Figure 9 shows DHAP anchored in both active sites. *Ec*Fuc-1PA had a medium-sized active site, with an upper hydrophobic pocket and two hydrophilic pockets, a lower one, necessary to retain the phosphate fragment by H bonds, and a lateral one. On the other hand, *Tm*Rhu-1PA had a substantially larger active site and an extra hydrophilic pocket, located in the upper left corner of the figure.

Through theoretical calculations using the molecular docking program, the interaction of *Tm*Rhu-1PA with the cytosine derivate **1b** was determined.

**Figure 10:**
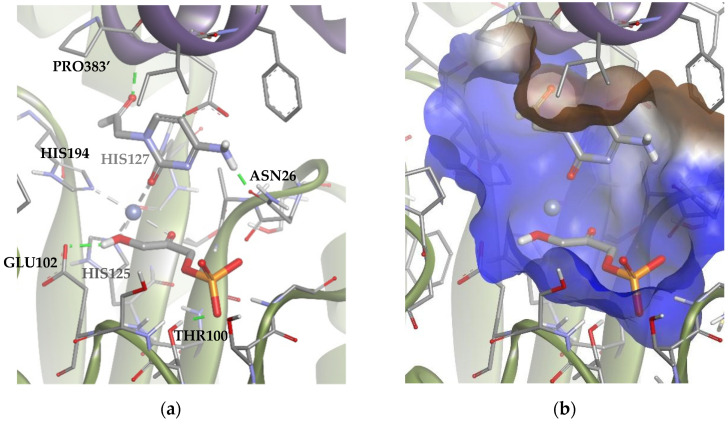
Docking for the cytosine derivative **1b** with *Tm*Rhu-1PA, generating Solution 1: (**a**) DHAP coordinated by the carbonyl group of **1b** ring, and (**b**) the -NH_2_ group of **1b** interacting with the lateral hydrophilic pocket and the aldehyde group of the derivate with the upper hydrophilic pocket of the active site. The interactions are represented as dashed lines: green (H-bonds) and grey (coordination bonds). In (**b**), hydrophobicity is mapped onto a Connolly solvent-accessible surface of the receptor. Non-polar hydrogen atoms are omitted for clarity. Atom color code: C (grey), N (blue), O (red), Zn/Co (violet), P (orange), and H (white). The two protein chains are depicted with different colors (green and turquoise).

All solutions obtained were similar to Solution 1 (Figure 10), where the substrate was coordinated by the carbonyl group of the ring, leaving the amino group to interact through an H-bond with ASN26 in the lateral hydrophilic pocket. Besides, the aldehyde group established another H-bond with PRO383′, located in the upper hydrophilic pocket. This pose allows the ring to establish significant Van der Waals interactions with the hydrophobic pocket.

**Figure 11:**
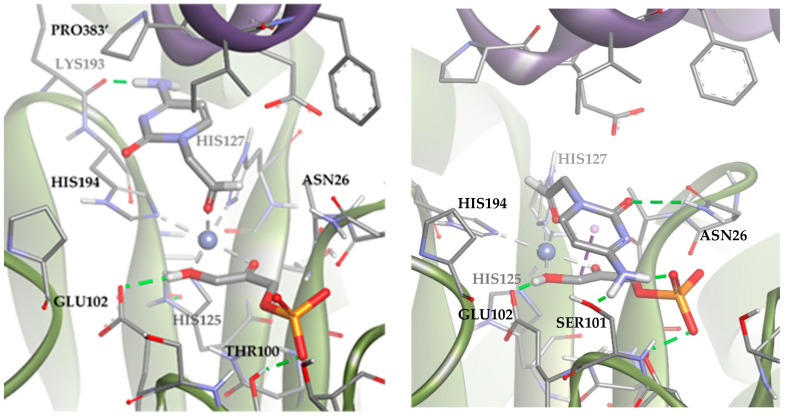

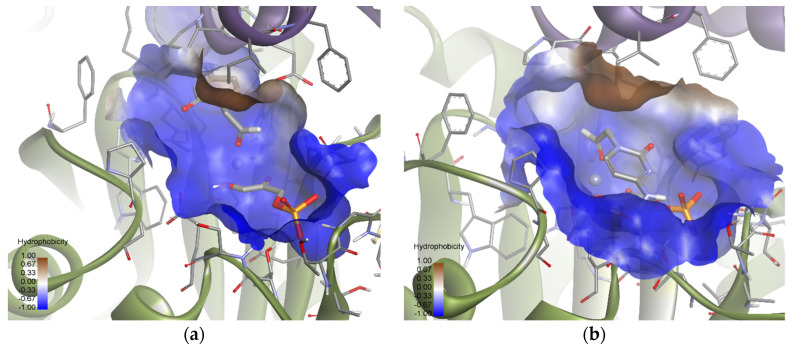
Docking for the cytosine derivative **1b** with *Tm*Rhu-1PA and DHAP generating two possible solutions: (**a**) Solution 2: the R group is directed toward the upper hydrophilic pocket and (**b**) Solution 3: the R group is directed toward the lower hydrophilic pocket. The interactions are represented as dashed lines: green (H-bonds), grey (coordination bonds), and pink (pi-mediated interactions). Hydrophobicity is mapped onto a Connolly solvent-accessible surface of the receptor. Non-polar hydrogen atoms are omitted for clarity. Atom color code: C (grey), N (blue), O (red), Co (violet), P (orange), and H (white). The two protein chains are depicted with different colors (green and turquoise).

These results suggested that the most stable poses of the substrate leave the aldehyde moiety far from the DHAP molecule. However, in the dynamic of the system, other poses compatible with the aldolic addition are expected to be populated, explaining the biocatalytic activity of the *Tm*Rhu-1PA. To study those conformations, an anchoring of the substrate was performed but adding a constriction to the distance between the oxygen of the aldehyde group and the metal ion (d = 1.5–3.5 Å; k = 5). The results are shown in Figure 11 (Solutions 2 and 3). The statistical relationship predicted by the model is 88:12 (3*R*,4*S*, L-*threo*):(3*R*,4*R*, D-*erythro*), in agreement with the experimental diastereomeric ratio of 75:25.

Solutions 2 and 3 mainly differed in the location of the R group of the aldehyde. In Solution 2, the R group was directed toward the upper hydrophilic pocket (typical of RhuA), establishing an H-bond with LYS93. Conversely, in the case of Solution 3, the R group was directed toward the lower hydrophilic pocket, interacting through hydrogen bonds with SER101, ASN26, and the DHAP phosphate group. An in-depth analysis of the factors contributing to the docking score (Table S2) revealed that Solution 2 is preferred over 3 because it maximizes Van der Waals substrate–protein interactions (see how the ligand interacts with the upper hydrophobic pocket only in Solution 2). However, Solution 3 keeps competing with Solution 2 from a statistical point of view, since it allows to increase the intensity of substrate–protein hydrogen bonds, but at the same time minimizes the clash with residues.

**Figure 12:**
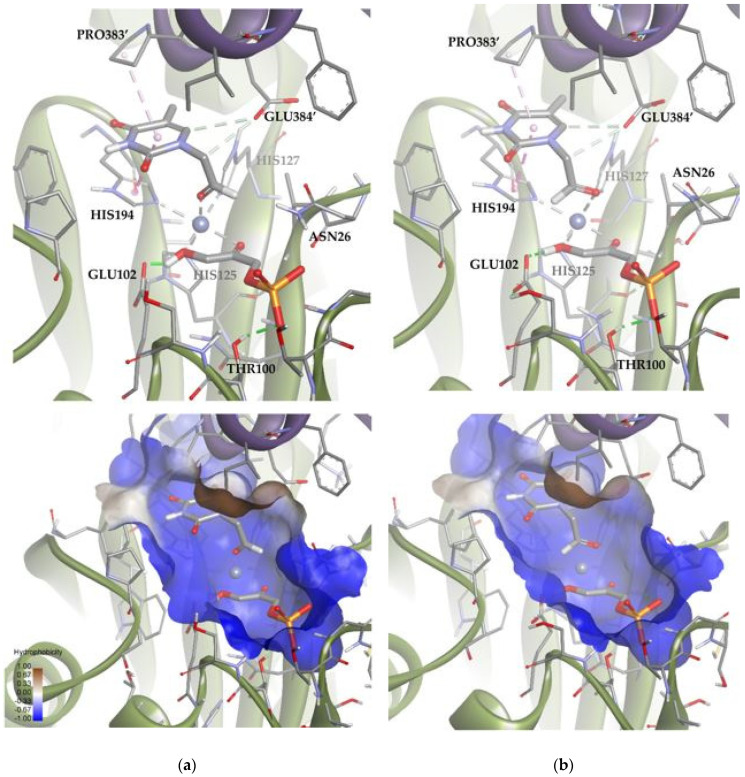
Docking for the thymine derivative **1a** with *Tm*Rhu-1PA: (**a**) Solution 4: the aldehydic hydrogen shifts its orientation, exposing the *re* face to the DHAP. (**b**) Solution 5: the aldehydic hydrogen exposes the *si* face to the DHAP. The interactions are represented as dashed lines: green (H-bonds), light green (non-conventional H-bonds), grey (coordination bonds), and pink (pi-mediated interactions). Hydrophobicity is mapped onto a Connolly solvent-accessible surface of the receptor. Non-polar hydrogen atoms are omitted for clarity. Atom color code: C (grey), N (blue), O (red), Zn/Co (violet), P (orange), and H (white). The two protein chains are depicted with different colors (green and turquoise).

A similar analysis was carried out with *Tm*Rhu-1PA and the thymine derivative **1a** as a substrate. The results for this system showed that 89 of the 100 solutions had the substrate bound to the metal ion through the aldehyde group (Figure 12). These solutions are, therefore, compatible with experimental aldol addition. Of those 89 solutions, in 80 the aldehydic hydrogen shifted its orientation, exposing the *re* face to the DHAP (Solution 4). The rest of the poses displayed the aldehyde group, exposing the *si* face to the DHAP (Solution 5). The calculated ratio was then 90:10, in agreement with the experimental product ratio (80:20). The preference of the substrate for the position of Solution 4 is not easy to explain, since in both docking alternatives, the substrate was bound to the active site through non-conventional H-bonds with GLU384′ and pi-mediated interactions with PRO383′ and HIS194. The structural difference seems to be the coordination scheme of the substrate to cobalt(II). When considering Solution 4, the distance between the oxygen of the carbonyl group and cobalt(II) increased 0.3 Å compared with Solution 5, causing a decrease in the strength of the coordination bond. Also, in Solution 4, the substrate presented slightly stronger hydrogen bonds with the active site (see Table S2).

**Figure 13:**
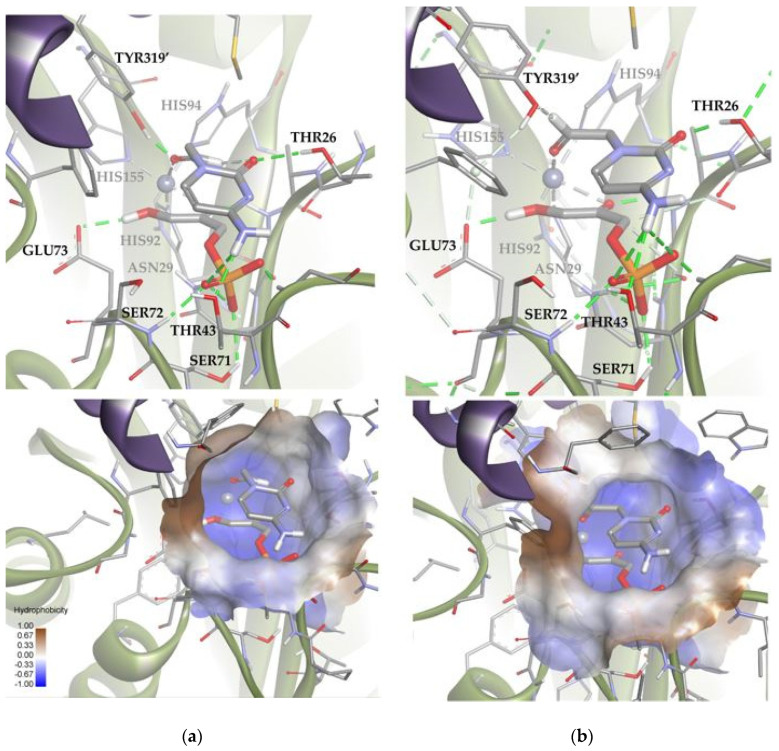
Docking for the cytosine derivative **1b** attached by the aldehyde group to the metal ion of the active site of *Ec*Fuc-1PA: (**a**) Solution 6: the aldehydic hydrogen shifts its orientation, exposing the *re* face to the DHAP. (**b**) Solution 7: the aldehydic hydrogen exposes the *si* face to the DHAP. The interactions are represented as dashed lines: green (H-bonds), light green (non-conventional H-bonds), grey (coordination bonds), and pink (pi-mediated interactions). Hydrophobicity is mapped onto a Connolly solvent-accessible surface of the receptor. Non-polar hydrogen atoms are omitted for clarity. Atom color code: C (grey), N (blue), O (red), Zn/Co (violet), P (orange), and H (white). The two protein chains are depicted with different colors (green and turquoise).

Subsequently, using the same molecular modeling methodology, the interaction of *Ec*Fuc-1PA with **1b** was determined and it showed highly variable docking solutions. Figure 13 shows a group of solutions that have the substrate attached to the metal ion of the active site by the aldehyde group. These solutions (about 10) are compatible with the aldol experimental reaction, since they locate the carbonylic carbon near C3 of DHAP. Within this group there are two subgroups: eight orienting the *re* face of the aldehyde moiety to the DHAP (Solution 6) and two the *si* face (Solution 7). These results are in agreement with the 70:30 (3*R*,4*S*, L-*threo*):(3*R*,4*R*, D-*erythro*) ratio of products observed experimentally.

Based on the calculations shown above (Figure 13 and Table S2), it is possible to draw some conclusions that explain the relative stability of both poses. Solutions 6 and 7 showed intense hydrogen bonds with both hydrophilic pockets, involving the residues TYR319′, THR26, THR43, and the DHAP’s phosphate group. However, Solution 6 was the one that allowed the generation of stronger hydrogen bonds without increasing the internal tension of the ligand and the clash with the protein. For this reason, this orientation is preferred over that corresponding to Solution 7.

**Figure 14:**
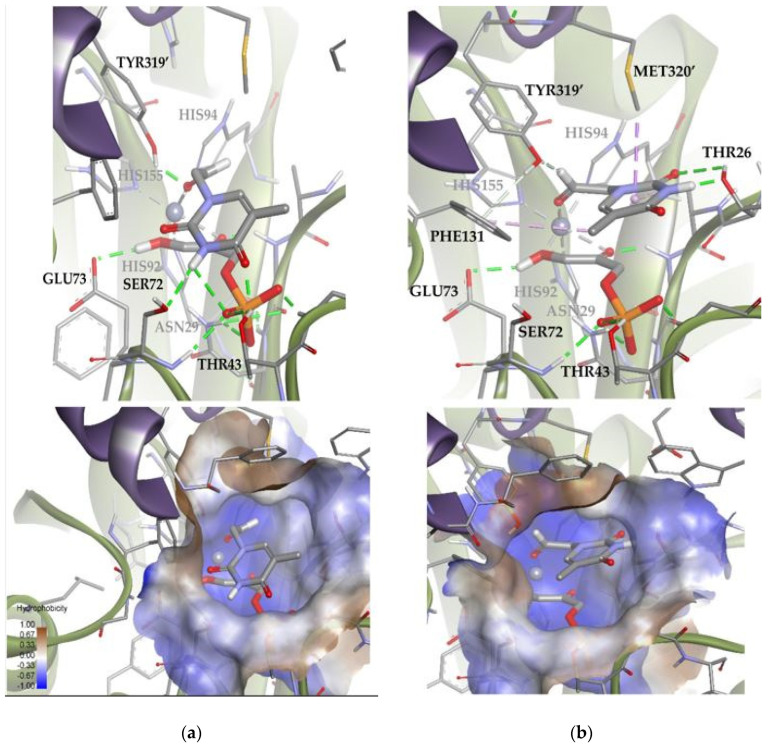
Docking for the thymine derivative **1a** attached by the aldehyde group to the metal ion of the active site of *Ec*Fuc-1PA: (**a**) Solution 8: Van der Waals interactions optimized in the hydrophobic pocket of the active site. (**b**) Solution 9: increase in the intensity of the substrate **1a**–protein hydrogen bonds while minimizing the steric clash. The interactions are represented as dashed lines: green (H-bonds), light green (non-conventional H-bonds), grey (coordination bonds), and pink (pi-mediated interactions). Hydrophobicity is mapped onto a Connolly solvent-accessible surface of the receptor. Non-polar hydrogen atoms are omitted for clarity. Atom color code: C (grey), N (blue), O (red), Zn/Co (violet), P (orange), and H (white). The two protein chains are depicted with different colors (green and turquoise).

A similar analysis was carried out with *Ec*Fuc-1PA and the thymine derivative **1a**. Among the 100 docking poses, one showed the substrate coordinated to the Zn^2+^ ion by the aldehyde group. This is indicative that there are a group of poses, less populated, compatible with the bio-catalyzed aldol addition. To gain insights into those conformations, the docking runs were repeated, adding a constraint to the distance between the oxygen of the aldehyde group and the metal ion (d = 1.5–3.5 Å; k = 5). Then, of 100 total solutions, 71 of them were similar to Solution 8 and the remaining 29 were similar to Solution 9 (Figure 14). These results are in agreement with the 75:25 (3*R*,4*S*, L-*threo*):(3*R*,4*R*, D-*erythro*) ratio observed by spectroscopic analysis. According to the docking results (Table S2), Solution 8 is more stable than 9 because it significantly optimizes Van der Waals interactions with the active site (strongly interacting with the hydrophobic pocket; Figure 14). In any case, Solution 9 is still competitive, since it allows to increase the intensity of the substrate–protein hydrogen bonds (involving the residues THR26 and TYR319′) while minimizing the steric clash.

## 4. Conclusions

This work described a green procedure for the preparation of new acyclic nucleoside analogues with potential antiviral activities.

In particular, the DHAP-dependent aldolase RAMA proved to be a very efficient enzyme for synthesizing monophosphorylated nucleoside analogues, a type of prodrug of proven usefulness in the field of nucleoside therapies. Due to its biochemical characteristics, RAMA allows the simultaneous introduction of two chiral centers and a phosphate group in a single synthetic step. Using DHAP and aldehyde derivatives of thymine and cytosine (**1a** and **1b**, respectively) as substrates, it was possible to obtain dihydroxyoxopentylphosphate derivatives with conversion between 70% and 90%. Through structural analysis by NMR, it was possible to verify the high diastereomeric purity of the products with configuration (3*S*,4*R*, D-*threo*), **2a** and **2b**, as predicted for this enzyme.

With the aim of searching for stereochemical diversity of AN, recombinant *Tm*Rhu-1PA and *Ec*Fuc-1PA were also tested. In this sense, when using *Tm*Rhu-1PA, conversion of 90% was obtained for **1a** and 86% for **1b**. In both cases, a major compound (**4a** and **4b**) with configuration (3*R*,4*S*, L-*threo*) and a minor product (**5a** and **5b**) whose stereochemistry was (3*R*,4*R*, D-*erythro*) were obtained. These results agree with previous bibliographic data on the enzyme. The diastereomeric ratios obtained were 80:20 for **4a**:**5a** and 75:25 for **4b**:**5b**.

On the other hand, when using *Ec*Fuc-1PA, high conversions were also obtained, 90% when **1a** was used as a substrate and 76.6% for **1b**. In this case, we were able to determine by NMR that the obtained diastereomeric ratio preserved the one that was found for *Tm*Rhu-1PA, being 75:25 for **4a**:**5a** and 70:30 for **4b**:**5b**, in contrast to what was expected according to the literature. Therefore, structural analyses by molecular modeling docking were carried out. The results obtained by these analyses were consistent with the experimental ones. It was observed that the stereochemistry of the obtained products strongly depends on the type of aldehydic substrate used in the aldol addition due to the different interactions of these with the active sites of each biocatalyst. Particularly, for *Ec*Fuc-1PA, in which the relationship between the stereoisomers obtained was inverse to that expected, molecular modeling allowed us to explain this fact.

In summary, new chiral acyclic analogues of pyrimidine nucleosides have been prepared using new substrates for DHAP-dependent aldolases, confirming their versatility in terms of the electrophilic electron-donor. The assessment of the biological activity of these compounds is in progress.

## Data Availability

The original contributions presented in the study are included in the article/Supplementary Material, further inquiries can be directed to the corresponding authors.

## Supplementary Materials

The following supporting information can be downloaded at: https://www.mdpi.com/article/10.3390/biom14070750/s1. Figure S1: ^1^H NMR of **2a**: D-*threo*-5-(1-thyminyl)-3,4-dihydroxy-2-oxopentyl phosphate and **3a**: L-*erytrho*-5-(1-thyminyl)-3,4-dihydroxy-2-oxopentyl phosphate, obtained with RAMA as a biocatalyst. Figure S2: ^13^C NMR of **2a** and **3a**: D-*threo* and L-*erythro*-5-(1-thyminyl)-3,4-dihydroxy-2-oxopentyl phosphate, obtained with RAMA as a biocatalyst. Figure S3: ^1^H NMR of **2b**: D-*threo*-5-(1-cytosyl)-3,4-dihydroxy-2-oxopentyl phosphate and **3a**: L-*erytrho*-5-(1-cytosyl)-3,4-dihydroxy-2-oxopentyl phosphate, obtained with RAMA as a biocatalyst. Figure S4: ^13^C NMR of **2b** and **3b**: D-*threo* and L-*erythro*-5-(1-cytosyl)-3,4-dihydroxy-2-oxopentyl phosphate, obtained with RAMA as a biocatalyst. Figure S5: ^1^H NMR of **4b**: L-*threo*-5-(1-cytosyl)-3,4-dihydroxydihydroxy-2-oxopentyl phosphate and **5b**: D-*erytrho*-5-(1-cytosyl)-3,4-dihydroxy-2-oxopentyl phosphate, obtained with *Tm*Rhu-1PA as a biocatalyst. Figure S6: ^13^C NMR of **4b** and **5b**: L-*threo* and D-*erythro*-5-(1-cytosyl)-3,4-dihydroxy-2-oxopentyl phosphate, obtained with *Tm*Rhu-1PA as a biocatalyst. Figure S7: ^1^H NMR of **4a**: L-*threo*-5-(1-thyminyl)-3,4-dihydroxy-2-oxopentyl phosphate and **5a**: D-*erytrho*-5-(1-thyminyl)-3,4-dihydroxy-2-oxopentyl phosphate, obtained with *Tm*Rhu-1PA as a biocatalyst. Figure S8: ^13^C NMR of **4a** and **5a**: L-*threo* and D-*erythro*-5-(1-thyminyl)-3,4-dihydroxy-2-oxopentyl phosphate, obtained with *Tm*Rhu-1PA as a biocatalyst. Figure S9: ^1^H NMR of **4a**: L-*threo*-5-(1-thyminyl)-3,4-dihydroxy-2-oxopentyl phosphate and **5a**: D-*erytrho*-5-(1-thyminyl)-3,4-dihydroxy-2-oxopentyl phosphate, obtained with *Ec*Fuc-1PA as a biocatalyst. Figure S10: ^13^C NMR of **4a** and **5a**: L-*threo* and D-*erythro*-5-(1-thyminyl)-3,4-dihydroxy-2-oxopentyl phosphate, obtained with *Ec*Fuc-1PA as a biocatalyst. Figure S11: ^1^H NMR of **4b**: L-*threo*-5-(1-cytosyl)-3,4-dihydroxy-2-oxopentyl phosphate and **5a**: D-*erytrho*-5-(1-cytosyl)-3,4-dihydroxy-2-oxopentyl phosphate, obtained with *Ec*Fuc-1PA as a biocatalyst. Figure S12: ^13^C NMR of **4b** and **5b**: L-*threo* and D-*erythro*-5-(1-cytosyl)-3,4-dihydroxy-2-oxopentyl phosphate, obtained with *Ec*Fuc-1PA as a biocatalyst. Table S1: DP4+ probability calculation for **2a** (D-*threo*) and **3a** (L-*erythro*). Table S2: Molecular modeling calculations for the interaction of *Ec*Fuc-1PA with **1b**.
