# Supplementary material for: Synthesis of Chiral Acyclic Pyrimidine Nucleoside Analogues from DHAP-Dependent Aldolases
Source: Biomolecules. 2024 Jun 25;14(7):750. doi: 10.3390/biom14070750 (PMC11274987; doi:10.3390/biom14070750)
Supplement: Supplementary file 1 [file biomolecules-14-00750-s001.zip › biomolecules-3049199-supplementary.pdf]

Supplementary Materials

Synthesis of chiral acyclic pyrimidine nucleoside analogues from DHAP-dependent aldolases

Mariano Nigro, Israel Sánchez-Moreno, Raúl Benito-Arenas, Ana Valino, Adolfo Iribarren, Nicolás Veiga, Eduardo García-Junceda and Elizabeth Lewkowicz

Contents

Supplementary Figure S1

Supplementary Figure S2

Supplementary Figure S3

Supplementary Figure S4

Supplementary Figure S5

Supplementary Figure S6

Supplementary Figure S7

Supplementary Figure S8

Supplementary Figure S9

Supplementary Figure S10

Supplementary Figure S11

Supplementary Figure S12

Supplementary Table S1

Supplementary Table S2

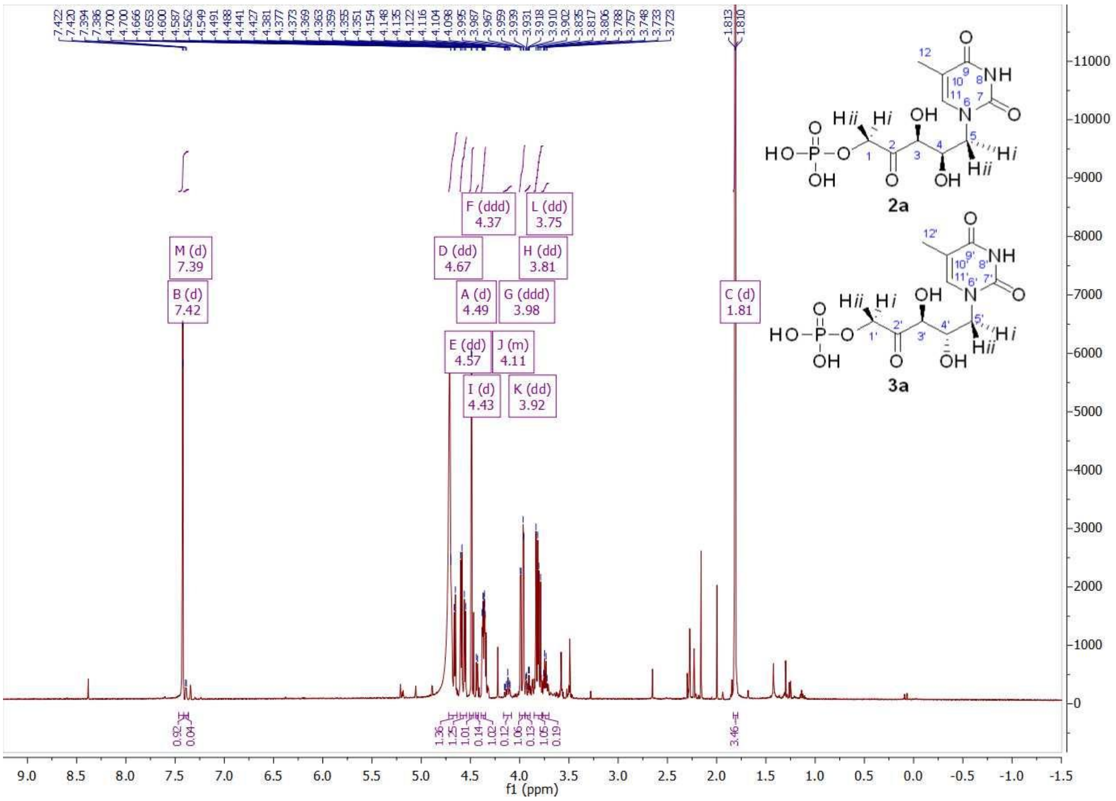

Figure S1: ^{1}H NMR analysis of **2a:** D-*threo*-5-(1-thyminyl)-3,4-dihyroxy-2-oxopentyl phosphate and **3a**: L-*erytrho*-5-(1-thyminyl)-3,4-dihyroxy-2-oxopentyl phosphate obtained with RAMA as biocatalyst.

2a ^{1}H NMR (D_2O, 500 MHz) δ (ppm) 7.42 (1H, d, *J* = 1.1 Hz, H-11), 4.67 (1H, dd, *J* = 18.5, 5,4 Hz, H-1*i*), 4.57 (1H, dd, *J* = 18.7, 6.6 Hz, H-1*ii*), 4.49 (1H, d, *J* = 1.9 Hz, H-3), 4.37 (1H, ddd, *J* = 8.9, 4.0, 1.9 Hz, H-4), 3.98 (1H, dd, *J* = 14.3, 4.0 Hz, H-5*i*), 3.81 (1H, dd, *J* = 14.2, 9.0 Hz, H-5*ii*) 1.81 (3H, d, *J* = 0.9 Hz, H-12).

3a ^{1}H NMR (D_2O, 500 MHz) δ (ppm) 7.39 (1H, d, *J* = 1.3 Hz, H-11), 4.67 (1H, dd, *J* = 18.5, 5,4 Hz, H-1*i*), 4.57 (1H, dd, *J* = 18.7, 6.6 Hz, H-1*ii*), 4.43 (1H, d, *J* = 6.9 Hz, H-3), 4.11 (1H, m, H-4), 3.92 (1H, dd, *J* = 14.3, 4.1 Hz, 5-H*ii*), 3.75 (dd, *J* = 14.2, 9.0 Hz, 5-H*i*), 1.81 (3H, d, *J* = 1.2 Hz, H-12).

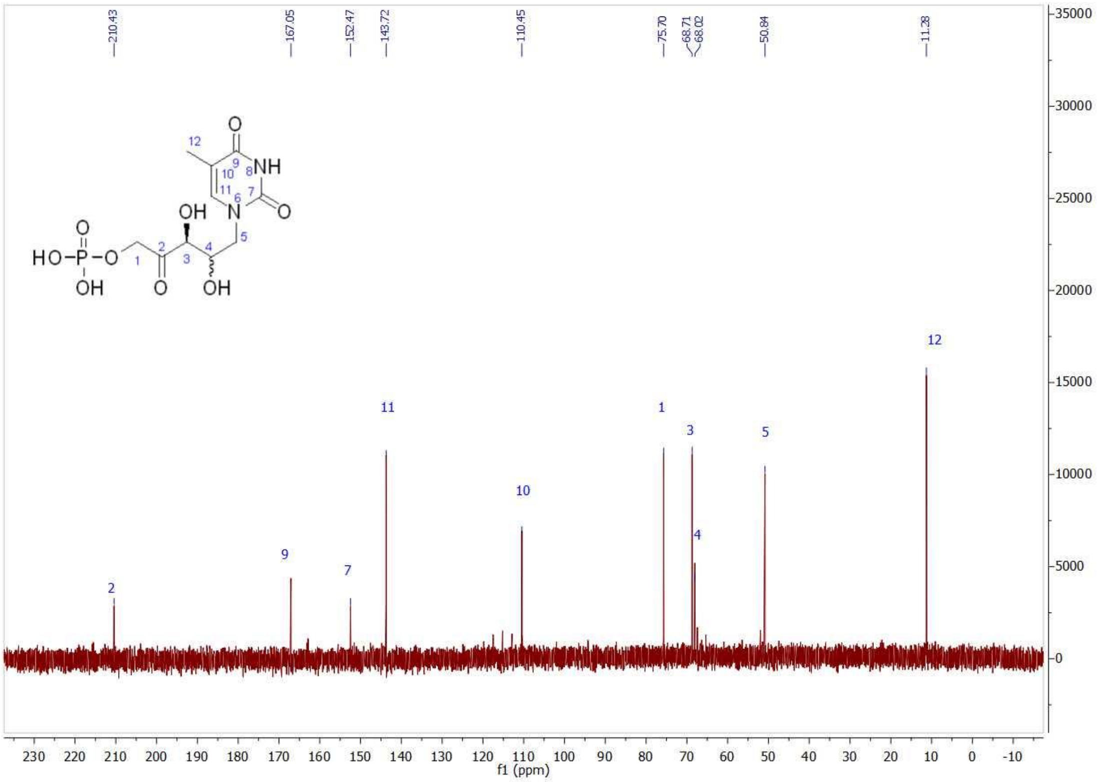

Figure S2: ^{13}C NMR analysis of **2a** and **3a:** D-*threo* and L-*erythro*-5-(1-thyminyl)-3,4-dihyroxy-2-oxopentyl phosphate obtained with RAMA as biocatalyst.

2a and **3a** ^{13}C NMR (D_2O, 125 MHz) δ (ppm): 210.43 (CO, C-2), 167.05 (C, C-9), 152.47 (C, C-7), 143.72 (CH, C-11), 110.45 (C, C-10), 75.70 (CH2, C-1), 68.71 (CHOH, C-3), 68.02 (CHOH, C-4), 50.84 (CH2, C-5), 11.28 (CH3, C-12).

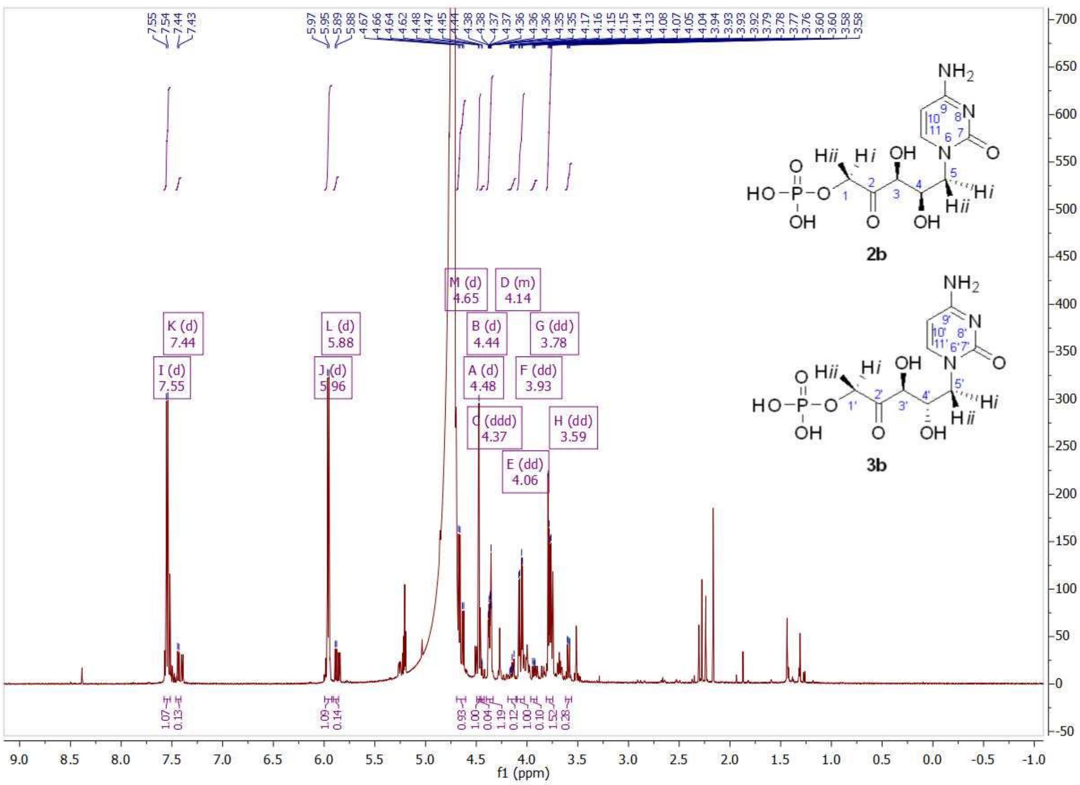

Figure S3: ^{1}H NMR analysis of **2b:** D-*threo*-5-(1-cytosyl)-3,4-dihyroxy-2-oxopentyl phosphate and **3b**: L-*erytrho*-5-(1-cytosyl)-3,4-dihyroxy-2-oxopentyl phosphate obtained with RAMA as biocatalyst.

2b ^{1}H NMR (D_2O, 500 MHz) δ (ppm) 7.55 (1H, d, *J* = 7.4 Hz, H-11), 5.96 (1H, d, *J* = 7.3 Hz, H-10), 4.65 (2H, dd, *J* = 18.6, 7.2 Hz, H-1*i*, *H-1ii*), 4.48 (1H, d, *J* = 2.0 Hz, H-3), 4.37 (1H, ddd, *J* = 8.2, 3.6, 1.5 Hz, H-4), 4.06 (1H, dd, *J* = 14.0, 4.0 Hz, H-5*i*), 3.78 (1H, dd, *J* = 14.3, 9.3 Hz, H-5*ii*).

3b ^{1}H NMR (D_2O, 500 MHz) δ (ppm) 7.44 (1H, d, *J* = 7.3 Hz, H-11), 5.88 (1H, d, *J* = 7.3 Hz, H-10), 4.65 (2H, dd, *J* = 18.6, 7.2 Hz, H-1*i*, H-1*ii*), 4.44 (1H, d, *J* = 3.2 Hz, H-3), 4.14 (1H, m, H-4), 3.93 (1H, dd, *J* = 14.0, 4.0 Hz, H-5*ii*), 3.59 (1H, dd, *J* = 15.1, 9.3 Hz, H-5*i*).

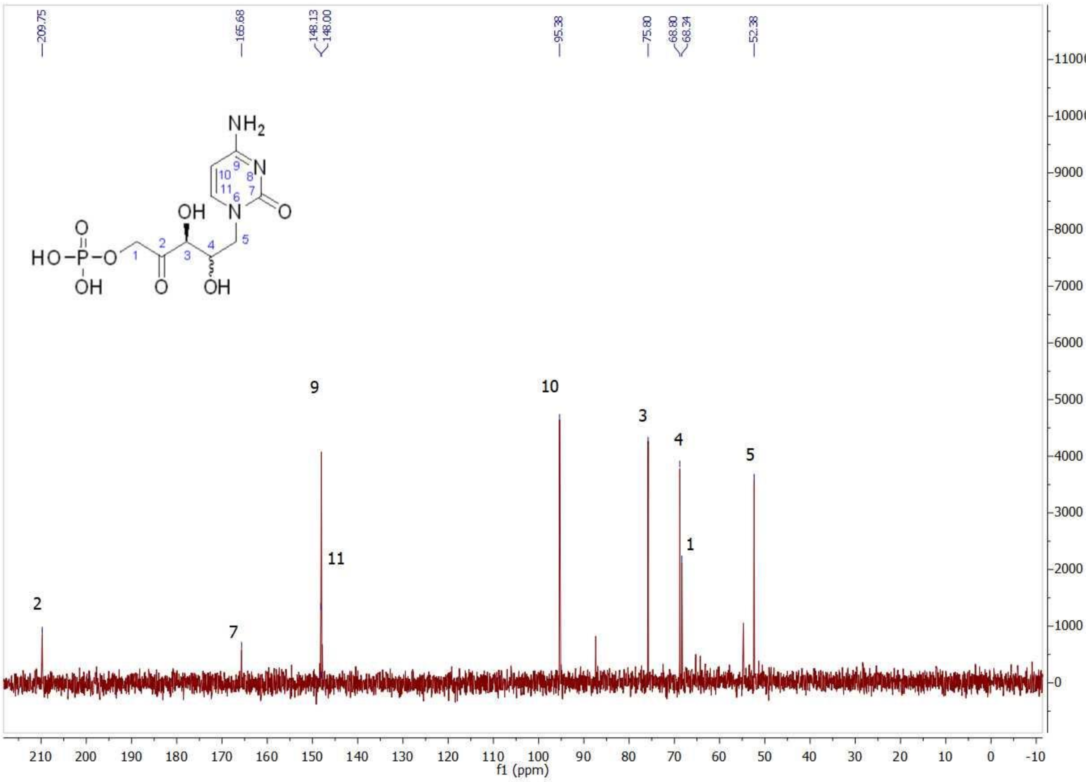

Figure S4: ^{13}C NMR analysis of **2b** and **3b:** D-*threo* and L-*erythro*-5-(1-cytosyl)-3,4-dihyroxy-2-oxopentyl phosphate obtained with RAMA as biocatalyst.

2b and **3b** ^{13}C NMR (D_2O, 125 MHz,) δ (ppm) 209.75 (CO, C-2), 165.68 (C, C-7), 148.13 C, C-9), 148.00 (CH, C-11), 95.38 (CH, C-10), 75.80 (CHOH, C-3), 68.80 (CHOH, C-4), 68.34 (CH2, C-1), 52.38 (CH2, C-5).

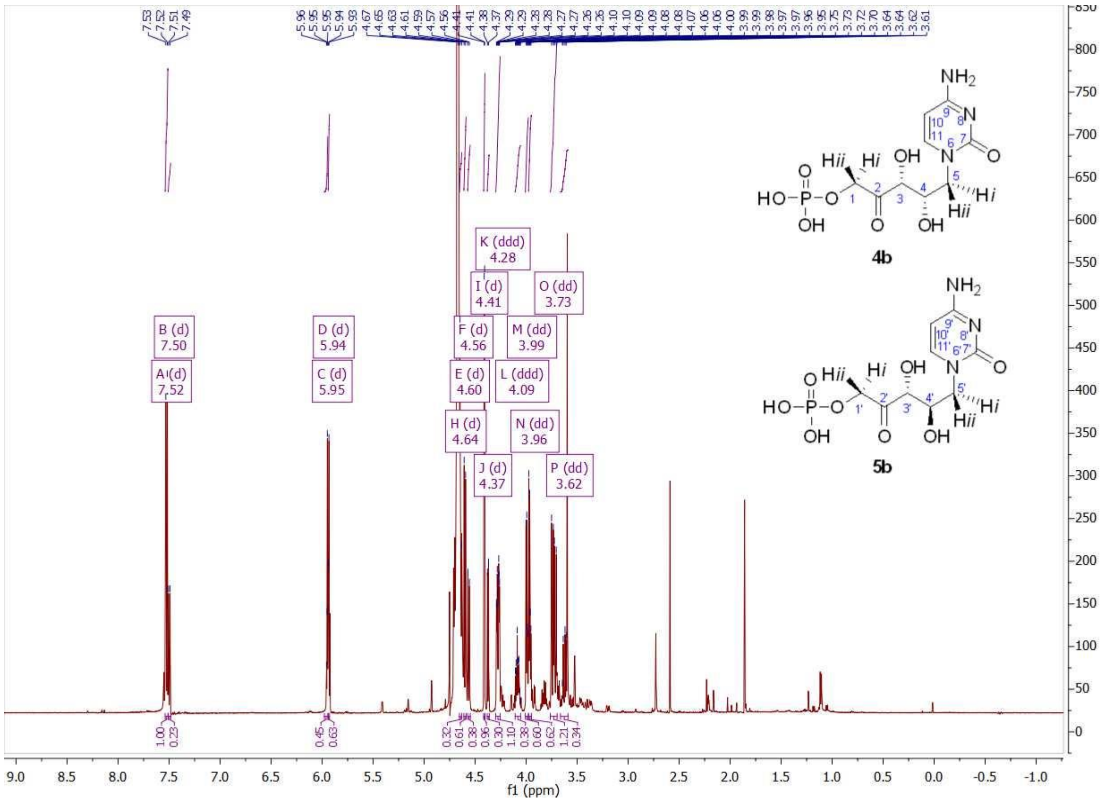

Figure S5: ^{1}H NMR analysis of **4b:** L-*threo*-5-(1-cytosyl)-3,4-dihyroxy-2-oxopentyl phosphate and **5b**: D-*erytrho*-5-(1-cytosyl)-3,4-dihyroxy-2-oxopentyl phosphate obtained with *Tm*Rhu-1PA as biocatalyst.

4b ^{1}H NMR (D_2O, 500 MHz) δ (ppm) 7.52 (1H, d, *J* = 7.4 Hz, H-11), 5.95 (1H, d, *J* = 7.4 Hz, H-10), 4.64 (2H, dd, *J* = 18.7, 7.3 Hz, H-1*i*, H-1*ii*), 4.60 (1H, dd, *J* = 18.7, 7.3 Hz, H-1*ii*), 4.41 (1H, d, *J* = 1.9 Hz, H-3), 4.28 (1H, ddd, *J* = 9.0, 3.9, 2.0 Hz, H-4), 3.99 (1H, dd, *J* = 14.1, 4.0 Hz, H-5*ii*), 3.73 (1H, dd, *J* = 14.1, 9.0 Hz, H-5*i*).

5b ^{1}H NMR (D_2O,500 MHz) δ (ppm) 7.50 (1H, d, *J* = 7.4 Hz, H-11), 5.94 (1H, d, *J* = 6.9 Hz, H-10), 4.64 (1H, dd, *J* = 18.7, 7.3 Hz, H-1*i*), 4.56 (1H, dd, *J* = 18.7, 7.3 Hz, H-1*ii*), 4.37 (1H, d, *J* = 4.7 Hz, H-3), 4.09 (1H, ddd, *J* = 8.8, 5.4, 3.2 Hz, H-4), 3.96 (1H, dd, *J*= 14.2, 3.2, H-5*i*), 3.62 (1H, dd, *J* = 14.3, 8.9 Hz, H5*ii*).

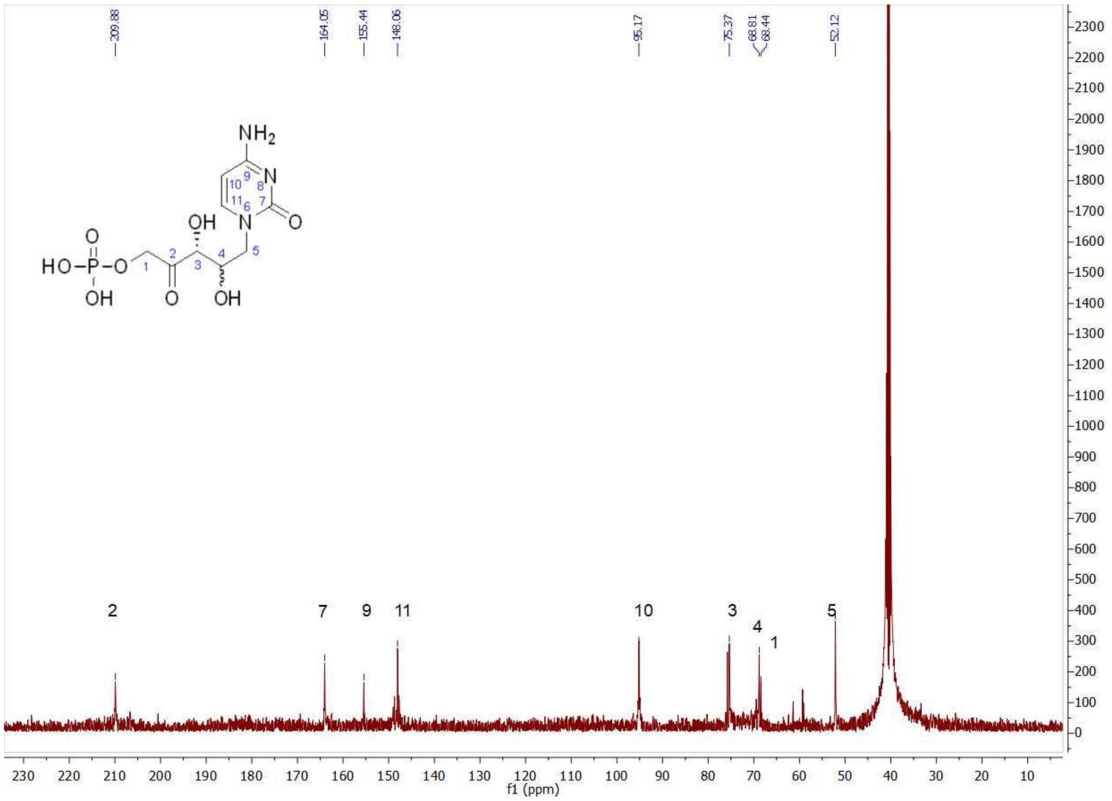

Figure S6: ^{13}C NMR analysis of **4b** and **5b:** L-*threo* and D-*erythro*-5-(1-cytosyl)-3,4-dihyroxy-2-oxopentyl phosphate obtained with *Tm*Rhu-1PA as biocatalyst.

4b and **5b** ^{13}C-NMR (D_2O, 125 MHz) δ (ppm): 209,88 (CO, C-2), 164,05 (CO, C-7), 155,44 (C, C-9), 148,06 (CH, C-11), 95,17 (CH, C-10), 75,37 (CHOH, C-3), 68,81 (CHOH, C-4), 68,44 (CH2, C-1), 52,12 (CH2, C-5).

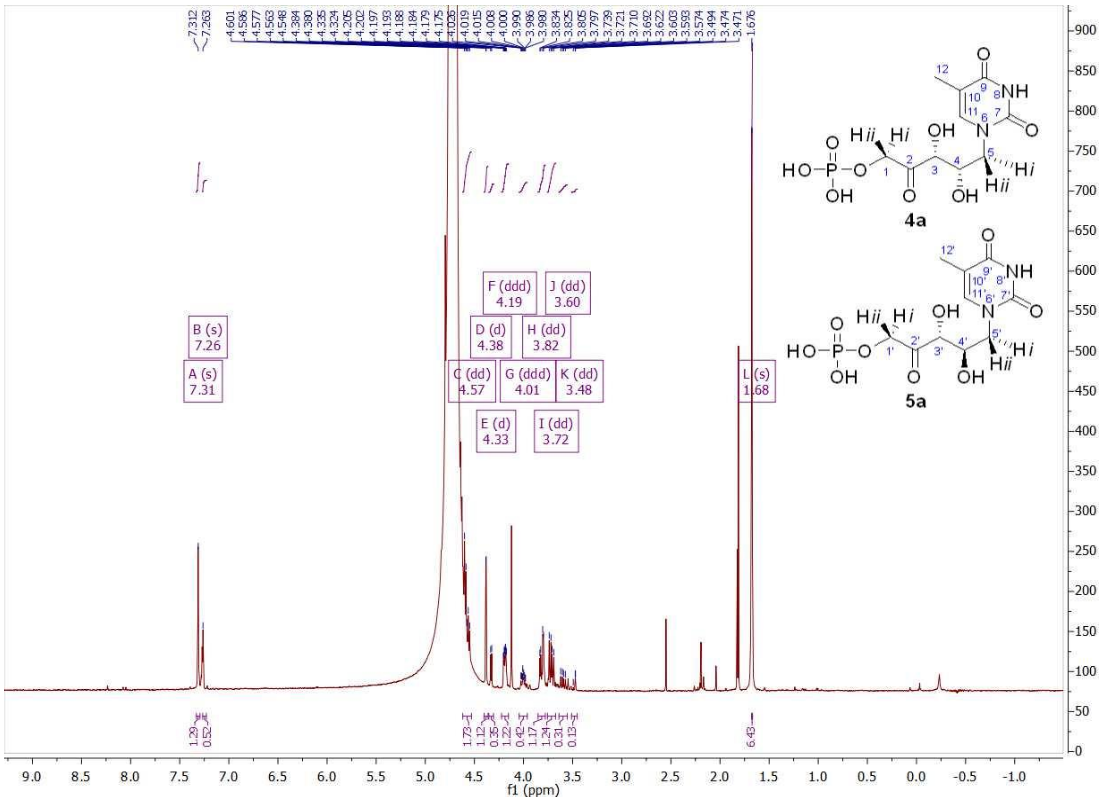

Figure S7: ^{1}H NMR analysis of **4a:** L-*threo*-5-(1-thyminyl)-3,4-dihyroxy-2-oxopentyl phosphate and **5a**: D-*erytrho*-5-(1-thyminyl)-3,4-dihyroxy-2-oxopentyl phosphate obtained with *Tm*Rhu-1PA as biocatalyst.

4a

^{1}H NMR (D_2O, 500 MHz) δ (ppm) 7.31 (1H, d, *J* = 0.9 Hz, H-11), 4.57 (2H, dd, *J* = 18.7, 7.4 Hz, H-1*i*, H-1*ii*), 4.38 (1H, d, *J* = 1.7 Hz, H-3), 4.19 (1H, ddd, *J* = 8.8, 4.2, 1.8 Hz, H-4), 3.82 (1H, dd, *J* = 14.3, 4.2 Hz, H-5*ii*), 3.72 (1H, dd, *J* = 14.3, 8.9 Hz, H-5*i*), 1.68 (3H, d, *J* = 0.8 Hz, H-12).

5a

^{1}H NMR (D_2O, 500 MHz) δ (ppm) 7.26 (1H, d, *J* = 0.9 Hz, H-11), 4.57 (2H, dd, *J* = 18.7, 7.4 Hz, H-1*i*, H-1*ii*), 4.33 (1H, d, *J* = 5.4 Hz, H-3), 4.01 (1H, ddd, *J* = 9.1, 5.2, 4.3 Hz, H-4), 3.60 (1H, dd, *J* = 14.5, 9.1 Hz, H-5*ii*), 3.48 (1H, dd, *J* = 14.0, 3.7 Hz, H-5*i*), 1.68 (3H, d, *J* = 0.8 Hz, H-12).

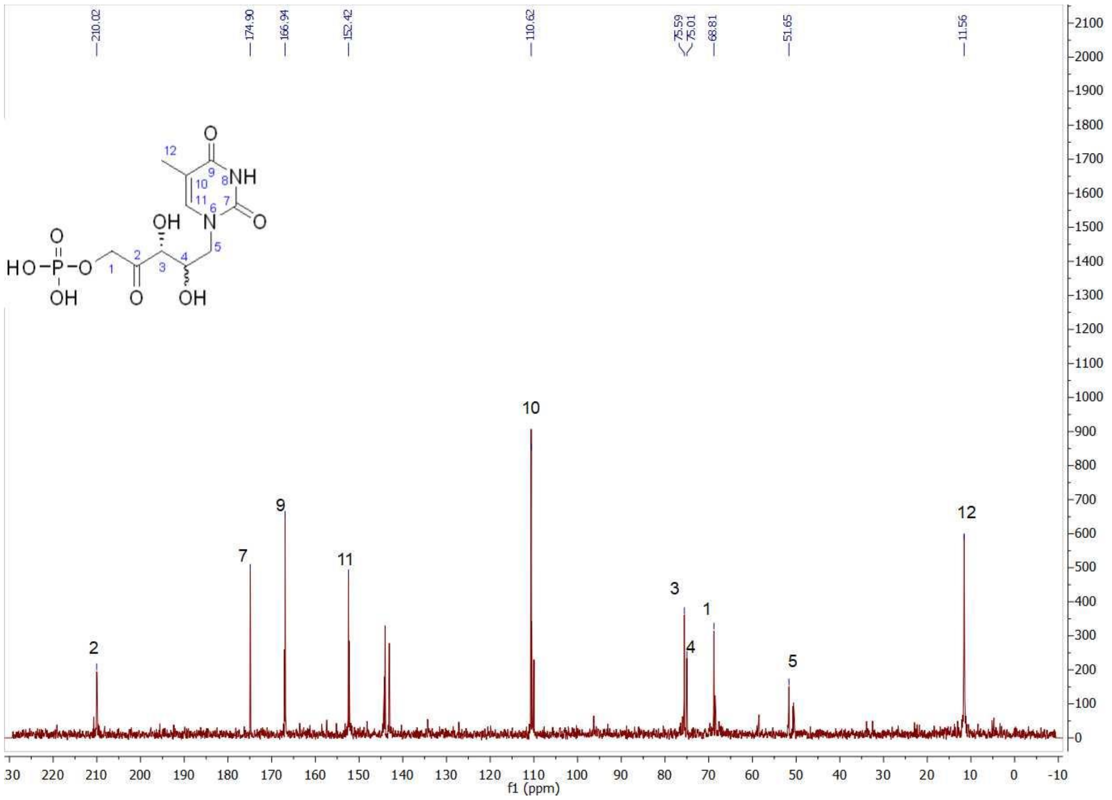

Figure S8: ^{13}C NMR analysis of **4a** and **5a:** L-*threo* and D-*erythro*-5-(1-thyminyl)-3,4-dihyroxy-2-oxopentyl phosphate obtained with *Tm*Rhu-1PA as biocatalyst.

4a and **5a** ^{13}C NMR (D_2O, 125 MHz) δ (ppm) 210.02 (CO, C-2), 174.90 (C, C-7), 166.94 (C, C-9), 152.42 (CH, C-11), 110.62 (C, C-10), 75.59 (CHOH, C-3), 75.01 (CHOH, C-4), 68.81 (CH2, C-1), 51.65 (CH2, C-5), 11.56 (CH3, C-12).

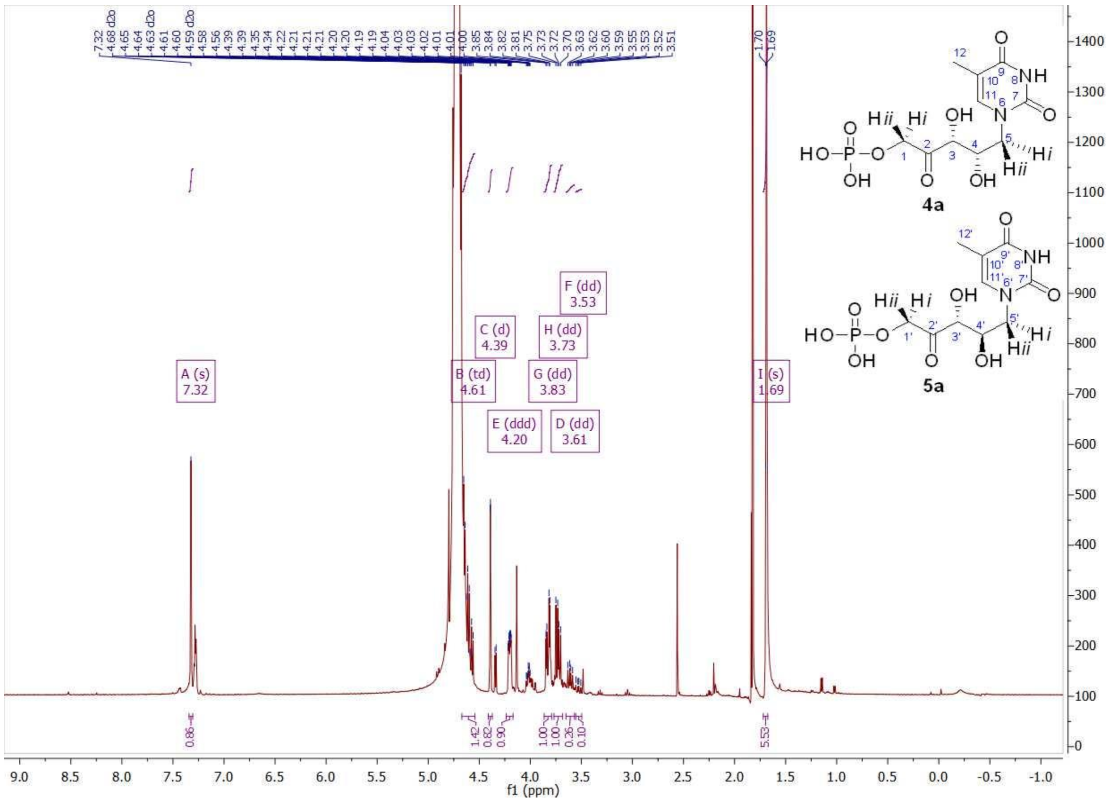

Figure S9: ^{1}H NMR analysis of **4a:** L-*threo*-5-(1-thyminyl)-3,4-dihyroxy-2-oxopentyl phosphate and **5a**: D-*erytrho*-5-(1-thyminyl)-3,4-dihyroxy-2-oxopentyl phosphate obtained with *Ec*Fuc-1PA as biocatalyst.

4a ^{1}H NMR (D_2O, 500 MHz) δ (ppm) 7.32 (1H, s ancho, H-11), 4.67 (1H, dd, H-1*i*), 4.59 (1H, dd, *J* = 18.6, 7.4 Hz, H-1*ii*), 4.39 (1H, d, *J* = 1.3 Hz, H-3), 4.20 (1H, ddd, *J* = 8.9, 3.9, 1.3 Hz, H-4), 3.83 (1H, dd, *J* = 14.3, 4.2 Hz, H-5*ii*), 3.73 (1H, dd, *J* = 14.3, 8.9 Hz, H-5*i*), 1.70 (3H, s, H-12).

5a ^{1}H NMR (D_2O, 500 MHz) δ (ppm) 7.32 (1H, s ancho, H-11), 4.67 (1H, dd, H-1*i*), 4.59 (1H, dd, *J* = 18.6, 7.4 Hz, H-1*ii*), 4.34 (1H, d, *J* = 5.4 Hz, H-3), 4.02 (1H, ddd, *J* = 8.7, 5.5, 3.1 Hz, H-4), 3.61 (1H, dd, *J* = 14.6, 9.0 Hz, H-5*ii*), 3.53 (1H, dd, *J* = 14.3, 4.3 Hz, H-5*i*), 1.69 (3H, s, H-12).

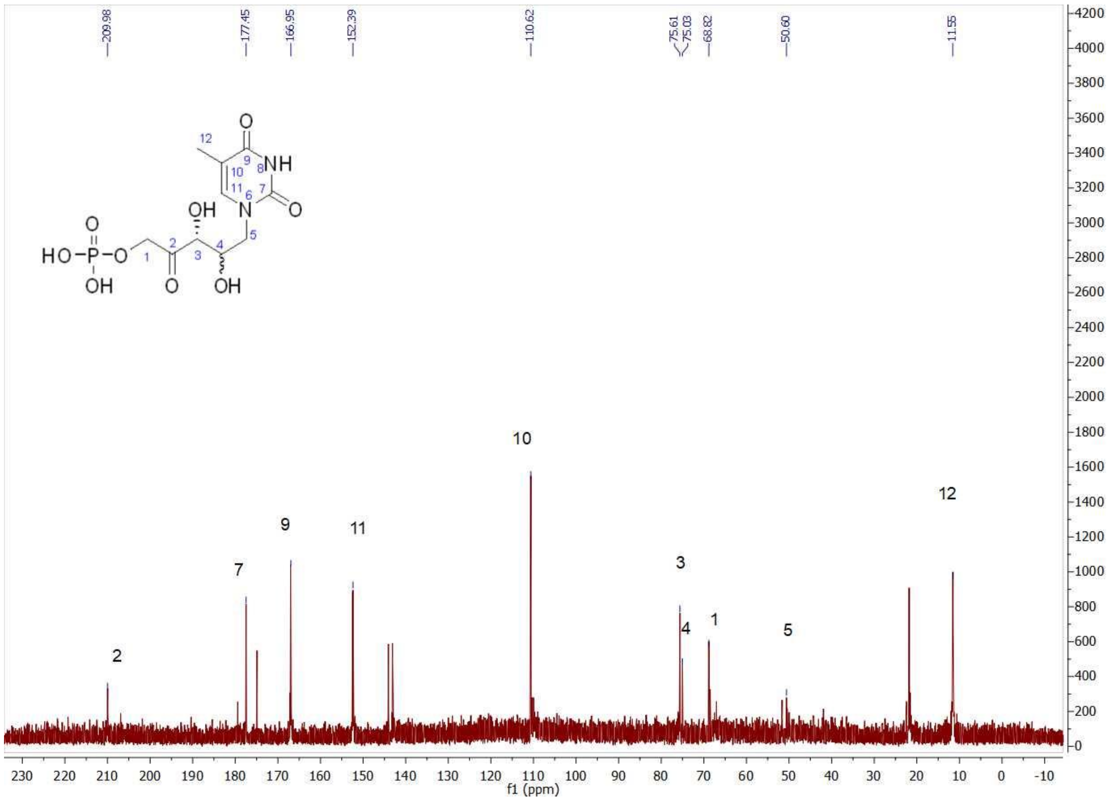

Figure S10: ^{13}C NMR analysis of **4a** and **5a:** L-*threo* and D-*erythro*-5-(1-thyminyl)-3,4-dihyroxy-2-oxopentyl phosphate obtained with *Ec*Fuc-1PA as biocatalyst.

4a and **5a** ^{13}C NMR (D_2O, 125 MHz,) δ (ppm) 209.93 (CO, C-2), 177.45 (C, C-7), 166.95 (C, C-9), 152.39 (CH, C-11), 110.62 (C, C-10), 75.61 (CHOH, C-3), 75.03 (CHOH, C-4), 68.82 (CH2, C-1), 50.60 (CH2, C-5), 11.55 (CH3, C-12).

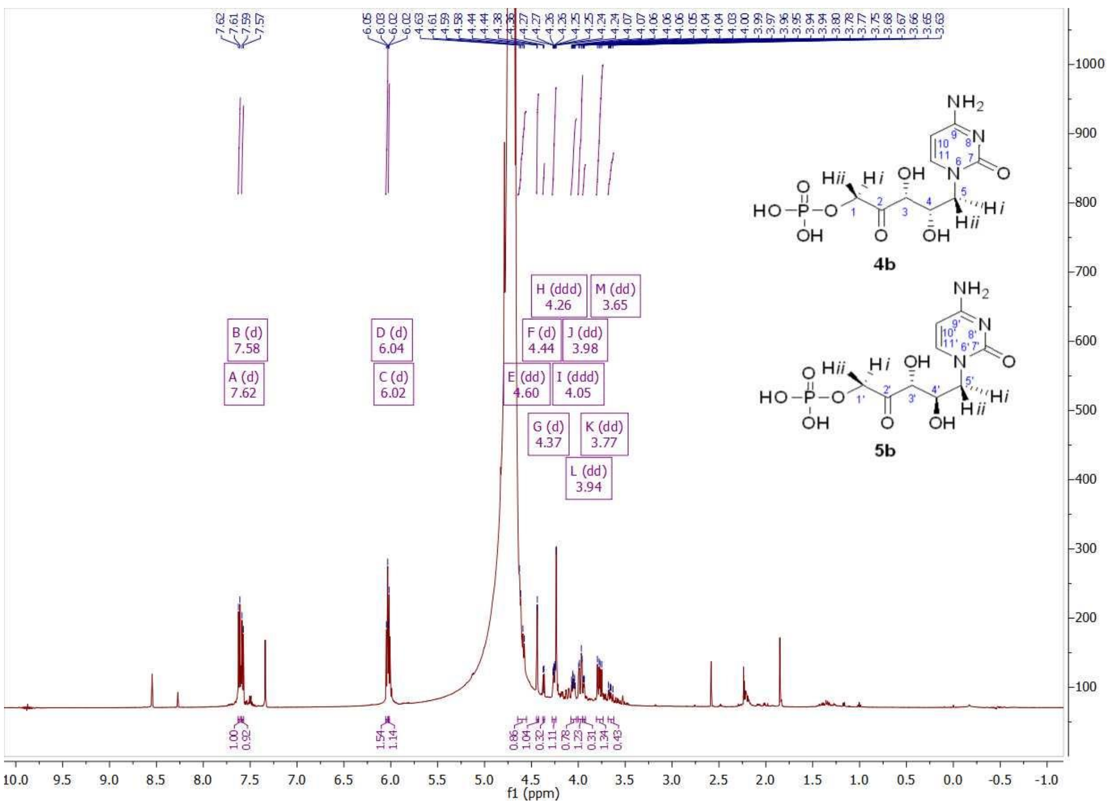

Figure S11: ^{1}H NMR analysis of **4b:** L-*threo*-5-(1-cytosyl)-3,4-dihyroxy-2-oxopentyl phosphate and **5a**: D-*erytrho*-5-(1-cytosyl)-3,4-dihyroxy-2-oxopentyl phosphate obtained with *Ec*Fuc-1PA as biocatalyst.

4b ^{1}H NMR (D_2O, 500 MHz) δ (ppm) 7.62 (1H, d, *J* = 7.4 Hz, H-11), 6.04 (1H, d, *J* = 7.4 Hz, H-10), 4.60 (1H, dd, *J* = 18.7, 7.3 Hz, H-1*i*), 4.44 (1H, d, *J* = 1.6 Hz, H-3), 4.26 (1H, ddd, *J* = 9.4, 3.7, 1.9 Hz, H-4), 3.98 (1H, dd, *J* = 14.1, 4.0 Hz, H-5*ii*), 3.77 (1H, dd, *J* = 14.1, 9.0 Hz, H-5*i*).

5b ^{1}H NMR (D_2O, 500 MHz) δ (ppm) 7.58 (1H, d, *J* = 7.4 Hz, H-11), 6.02 (1H, d, *J* = 6.9 Hz, H-10), 4.60 (1H, dd, *J* = 18.7, 7.3 Hz, H-1*i*), 4.37 (1H, d, *J* = 5.5 Hz, H-3), 4.05 (1H, ddd, *J* = 8.4, 5.6, 3.5 Hz, H-4), 3.94 (1H, dd, *J*= 14.2, 3.2 Hz, H-5*i*), 3.65 (1H, dd, *J* = 14.3, 8.9 Hz, H-5*ii*).

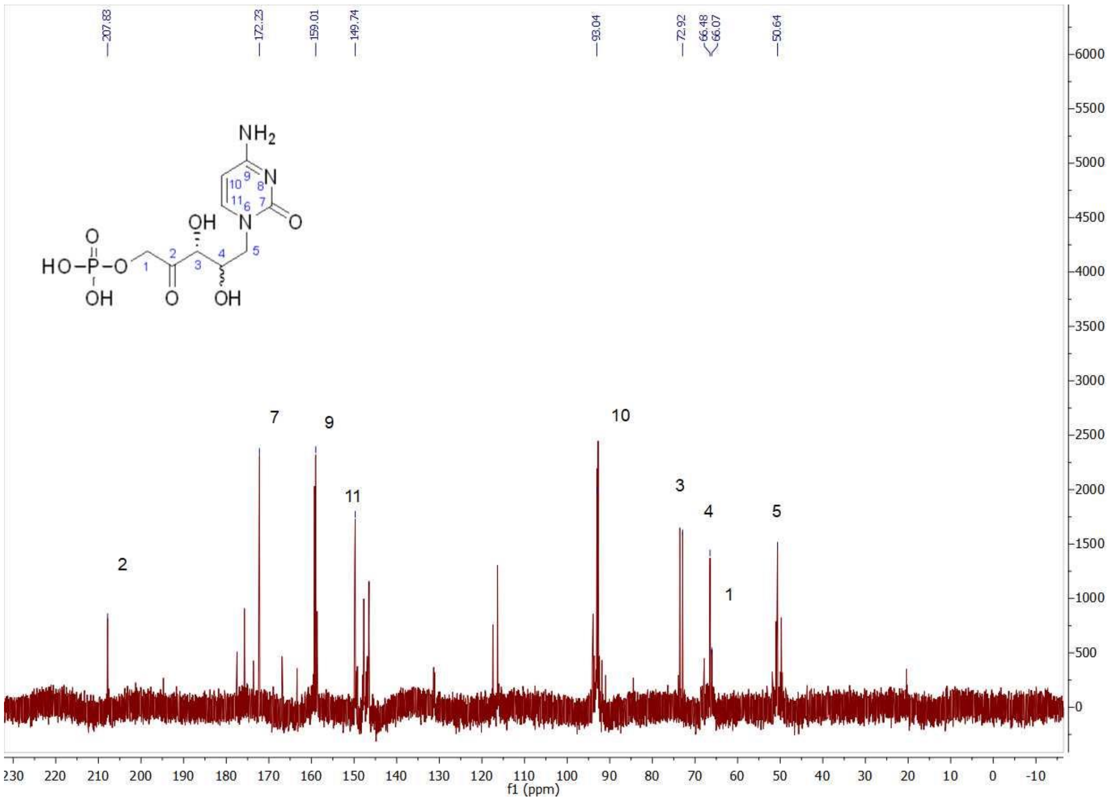

Figure S12: ^{13}C NMR analysis of **4b** and **5b:** L-*threo* and D-*erythro*-5-(1-cytosyl)-3,4-dihyroxy-2-oxopentyl phosphate obtained with *Ec*Fuc-1PA as biocatalyst**.**

4b and **5b** ^{13}C NMR (D_2O, 125 MHz) δ (ppm) 207.86 (CO, C-2), 172.23 (CO, C-7), 159.01 (C, C-9), 149.74 (CH, C-11), 93.04 (CH, C-10), 72.92 (CHOH, C-3), 66.48 (CHOH, C-4), 66.07 (CH2, C-1), 50.64 (CH2, C-5).

Table S1: DP4+ probability calculation for **2a** (D-*threo*) and **3a** (L-*erythro*).

	**D-*threo* (2a)**	**L- *erytro*(3a)**
**sDP4+ (H data)**	100.00%	0.00%
**sDP4+ (C data)**	99.99%	0.01%
**sDP4+ (all data)**	100.00%	0.00%
**uDP4+ (H data)**	99.99%	0.01%
**uDP4+ (C data)**	99.50%	0.50%
**uDP4+ (all data)**	100.00%	0.01%
**DP4+ (H data)**	100.00%	0.00%
**DP4+ (C data)**	100.00%	0.00%
**DP4+ (all data)**	100.00%	0.00%

Table S2: Molecular docking results.

Protein		*Tm*Rhu-1PA				*Ec*Fuc-1PA			
Substrate		**1b**		**1a**		**1b**		**1a**	
		Solutions		Solutions		Solutions		Solutions	
**Parameter**	Description	**2**	**3**	**4**	**5**	**6**	**7**	**8**	**9**
**Goldscore**	Total GoldScore fitness value of docked ligand	33.8	30.3	38.2	37.4	27.9	25.4	23.6	22.4
**External.Hbond**	Protein-ligand H-bond contribution to GoldScore value	12.2	13.4	9.97	9.43	13.7	12.9	11.3	13.0
**External.Vdw**	Protein-ligand vdw contribution to GoldScore value	34.9	26.1	38.6	38.9	24.8	26.3	26.5	21.1
**Internal.Vdw**	Internal ligand vdw contribution to GoldScore value	-0.06	0.37	0.35	0.17	-1.38	-1.06	-0.81	-2.26
**Internal.Torsion**	Internal ligand torsion-strain contribution to GoldScore value	-1.54	-1.13	-1.69	-1.65	-1.15	-2.67	-2.33	-1.79
**Protein.Energy**	Protein energy term to penalize clashes when using flexible sidechains	-10.16	-7.74	-9.06	-9.43	-8.09	-10.01	-9.20	-6.81
